# An Update on the Clinical Efficacy and Safety of Collagen Injectables for Aesthetic and Regenerative Medicine Applications

**DOI:** 10.3390/polym15041020

**Published:** 2023-02-17

**Authors:** Luca Salvatore, Maria Lucia Natali, Chiara Brunetti, Alessandro Sannino, Nunzia Gallo

**Affiliations:** 1 Typeone Biomaterials S.r.l., Muro Leccese, 73100 Lecce, Italy; 2 Department of Engineering for Innovation, University of Salento, 73100 Lecce, Italy

**Keywords:** collagen, injectable collagen, medical devices

## Abstract

Soft tissues diseases significantly affect patients quality of life and usually require targeted, costly and sometimes constant interventions. With the average lifetime increase, a proportional increase of age-related soft tissues diseases has been witnessed. Due to this, the last two decades have seen a tremendous demand for minimally invasive one-step resolutive procedures. Intensive scientific and industrial research has led to the recognition of injectable formulations as a new advantageous approach in the management of complex diseases that are challenging to treat with conventional strategies. Among them, collagen-based products are revealed to be one of the most promising among bioactive biomaterials-based formulations. Collagen is the most abundant structural protein of vertebrate connective tissues and, because of its structural and non-structural role, is one of the most widely used multifunctional biomaterials in the health-related sectors, including medical care and cosmetics. Indeed, collagen-based formulations are historically considered as the “gold standard” and from 1981 have been paving the way for the development of a new generation of fillers. A huge number of collagen-based injectable products have been approved worldwide for clinical use and have routinely been introduced in many clinical settings for both aesthetic and regenerative surgery. In this context, this review article aims to be an update on the clinical outcomes of approved collagen-based injectables for both aesthetic and regenerative medicine of the last 20 years with an in-depth focus on their safety and effectiveness for the treatment of diseases of the integumental, gastrointestinal, musculoskeletal, and urogenital apparatus.

## 1. Introduction

**Figure 1:**
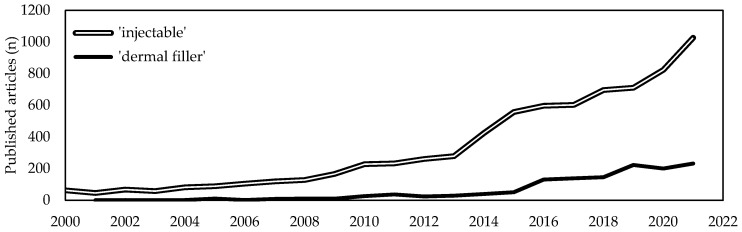
The increasing research interest in injectable formulations and dermal filler. Articles indexed in Scopus (www.scopus.com) with the keywords ‘injectable’ and ‘dermal filler’ and published from 2000 to 2022 (last accessed on 27 May 2022).

Soft tissues loss could be due to iatrogenic, traumatic, pathological, or physiological reasons. Aside from significantly affecting patients’ quality of life, their surgical management requires targeted, costly and sometimes constant interventions. With the average life increase, a proportional increase of age-related soft tissues diseases has been witnessed. Due to this, recent decades have seen a tremendous demand for soft tissue reconstruction strategies and one step resolutive procedures. Intense scientific and industrial research has been conducted to develop innovative approaches or optimize current solutions. Among them, in the last two decades injectable formulations have attracted even more interest for both aesthetic and regenerative surgery for their versatility and multifunctionality (Figure 1). Indeed, injectable scaffolds could be used in large and irregularly shaped lesions for a huge variety of damaged tissues, as well as providing temporary pain relief and functional improvement with a single treatment. Thus, injectable formulations could reduce the number of surgical procedures, costs, times and accelerate healing rate and quality.

The popularity of minimally invasive techniques increased rapidly for several reasons. A principal factor is the acceptance of soft tissue fillers among patients that are not ready for permanent treatments [1]. In the case of patients not wishing to undergo surgery, an easier procedure would generally be more accepted. Moreover, compared to undergoing more invasive surgery, fillers offer the patient less discomfort and a shorter recovery time, making them very practical in the resolution of minor-serious disease and allowing patients to return immediately to their daily routine [1,2]. Minimally invasive therapies would give a better quality of life also for that part of population that would otherwise not survive the trauma induced by conventional surgeries. Moreover, they could delay the execution of invasive surgical procedures for the implantation of permanent devices [3]. In the case of a staged surgical intervention, the use of injectable systems may avoid the need for multiple invasive operations, thus reducing the related morbidities and negative aesthetic effects associated with repeated procedures [4]. With regard to aesthetic treatments, minimally invasive therapies are preferred as they are less impacting and give a more natural look. Moreover, the lack of an external incision or an autologous tissue donor site is preferred because the absence of scarring is usually socially and psychologically more accepted.

From the surgeon’s point of view, the advantages of minimally invasive procedures include principally the need for fewer resources (e.g., operating room, staff, equipment, and time). Being simpler, transcutaneous injections require less operating room staff and time. The pro-regenerative action of injectables would reduce operating room time also because they would be able to restore physiological conditions with a single injection. However, it should not be forgotten that simpler procedures are not less exhausting and do not require less experience. Like any surgical procedure, minimally invasive therapies require adequate knowledge in order to reach the best outcome and avoid unwanted adverse events. 

Thus, not only clinicians’ but especially patients’ preference for fewer invasive and expensive procedures has undoubtedly promoted their use [4,5,6,7,8,9,10]. An injectable formulation for soft tissues reconstruction currently relies on two main approaches, involving autologous tissue displacement (e.g., lipofilling, platelet-rich plasma) or biomaterials-based filling [5]. Both approaches have some advantages and drawbacks. Autologous materials provide the most physiological solution (no adverse events or immune reactions) but suffer from donor site morbidity, volume resorption rate variability, and double surgery requirements. Moreover, their harvesting is a time-consuming procedure that requires double intervention. Alternatively, biomaterials offer an off-the-shelf solution with immediate results and should be distinguished as non-resorbable and resorbable, depending on their half-life. Non-resorbable solutions (e.g., silicone, poly(methyl methacrylate), polyvinylpyrrolidone, polyacrylamide), are permanent (last more than 2 years) but usually suffer from mild-severe adverse reactions (i.e., granuloma, implant encapsulation, persistent pain or rejection) that limit patient satisfaction and could require implant removal surgery [6,7,8,9,10,11,12]. Contrarily, resorbable formulations are usually based on natural biomaterials (i.e., collagen, hyaluronic acid, calcium hydroxyl apatite) and last 6–18 months [13,14,15,16]. Their durability depends on many factors such as the raw material type, product cross-linking degree, lost tissue extension, disease site and etiology, and patient metabolism, age and co-morbidities. The most used resorbable dermal fillers are collagen or hyaluronic acid based. 

Collagen is the most abundant structural protein of vertebrate connective tissues [17,18,19,20,21,22,23,24,25] and plays a crucial structural role for the maintenance of tissues’ architecture, shape and mechanical properties [20]. Moreover, by mediating a fundamental inter- and intracellular signaling it dictates specialized regulatory functions, especially during development and repair processes [26,27,28,29,30,31,32]. Type I collagen is one of the most widely used biomaterials in the health-related sectors, including medical care and cosmetics [17,18,19,20,21,22,23,24,25,33]. Several collagen-based injectable products have been approved for clinical use and used in many clinical settings.

This review will specifically focus on the clinical efficacy of collagen-based injectables for both aesthetic and regenerative medicine from 2000 until today. In particular, collagen extraction sources for injectables development and relative applications are discussed. To the best of our knowledge, we collected and discussed all pertinent research reports, commercial products data, and clinical trials about approved collagen-based injectable formulations, in order to underline the advantages and disadvantages related to their use. Accordingly, the available clinical results of the last 20 years about some of the leading collagen-based approved products were gathered and discussed according to the body site and pathology. In particular, this review focused on collagen-based injectables currently used for the regeneration of the musculoskeletal, urogenital, gastrointestinal, and integumental systems as well as for non standard clinical applications by presenting exemplary attempts to improve tissues’ regenerative performance. Finally, collagen-based products adverse events rate and their regulation are discussed.

## 2. Methodology

A deep search was undertaken on studies about injectable collagen alone or in combination with other materials for cosmetic and medical applications. The electronic search engines used were PubMed (https://pubmed.ncbi.nlm.nih.gov, accessed on 4 January 2023), ScienceDirect (https://www.sciencedirect.com, accessed on 4 January 2023), Google Scholar (https://scholar.google.com, accessed on 4 January 2023) and U.S. National Library of Medicine (https://clinicaltrials.gov/, accessed on 22 December 2022). The keywords used were ‘injectable’ and ‘collagen’. Several synonyms were searched for each component (i.e., injection, hydrolysate, gelatin, dermal filler, solution, colloid, infusion, hydrogel). The search included all studies related to injectable collagen-based formulations, including clinical trials, prospective case series, retrospective reviews, and case reports, independently from their level of evidence. A total of 125 studies were screened from 2000 to 2022 and reviewed.

## 3. Collagen as Biomaterial

Collagen is the most abundant structural protein of vertebrate connective tissues, and accounts for about the 30% of the total body protein content [17,18,19,20,21,22,23,24,25]. The collagen family is a group of proteins that share a unique molecular fingerprint that is characterized by the presence of a right-handed triple-helical domain formed by three left-handed polyproline-II helices [26,34,35]. This superfamily accounts for 28 members, named from type I to XXVIII according to the discovery order [34,36]. Type I collagen was the first to be discovered and accounts for the 70% of the total collagen found in the human body [26]. This protein is a hetero trimer of about 400 kDa consisting of two identical α1 (≈139 kDa) chains and one α2 (≈129 kDa) chain of about 1000 amino acid residues [20,37]. Both chains are characterized by the repetition of the Glycine-X-Y triplet, where the X and Y positions are usually represented by proline and hydroxyproline, respectively [34,37]. Hydroxylation of proline residues is a typical modification of collagen and, because it accounts for about 11–14% of total residues, it is commonly used as a marker to detect and quantify collagen in tissues [35,38]. Another peculiarity of fibril-forming type I collagen molecules is their ability to spontaneously assemble to form fibrils in which molecules are quasi-hexagonally packed and super-twisted in a right-handed structure along the longitudinal axis of the fibril [39,40,41]. Thus, collagen molecules are aligned parallel to one another with a staggering of about 67 nm (D-banding) and can assemble into fibrils that can be greater than 500 µm in length and 500 nm in diameter [25,34,42,43]. Then, fibrils assemble in fibers whose 3D arrangement is tissue specific.

Type I collagen not only covers a crucial structural role in tissue architecture maintenance but is actively involved in several biological and pathological processes [44]. The involvement of collagen in numerous cellular processes prompted research towards the use of collagen as biomaterial for the development of simplified ECM-like structures [20,35]. To this, several companies isolate medical-grade type I collagen from several sources (Table 1) and manufacture collagen-based implantable devices that are currently used in many clinical settings. Besides its advantages in term of biocompatibility for its physiological structural and non-structural functions, the use of collagen as biomaterial offers several advantages including low immunogenicity, tunable properties, and biodegradability. The low evolutionary gap and the high conservation of type I collagen amino acid composition among vertebrates make that homology up to 95% [19,45,46,47,48].

The possibility to define specific scaffolds properties (i.e., by tuning protein concentration, solvent type and concentration, protein molecular weight, superficial morphology, 3D organization, and by pre- and post-production processing) offers a great opportunity to modulate the structure-related biological activity of the scaffolds in order to optimize their capability to induce and sustain tissue regeneration [20,35,43,49,50,51,52,53,54,55]. Moreover, the use of collagen is advantageous for regenerative medicine and tissue engineering applications because, being recognized as a self-molecule, it is metabolized by the natural body enzymatic apparatus that gradually breaks down collagen molecules and substitutes it with newly synthetized one. Molecular pathways that mediate collagen degradation are several and are tissue and cell specific [39]. In general, the human body has several collagen degrading enzymes including the matrix metalloproteinases (in particular, matrix metalloproteinase 1), and cathepsins and neutrophil elastase that cleave collagen molecules which undergo a successive proteolytic process that depends on several factors (i.e., triple helix stability, protein amino acid sequence, crosslinking) [56,57,58,59,60,61]. Generally, collagen fragments resulting from the action of collagenases are further degraded by gelatinases and non-specific proteases. Thus, the presence of an accurate and complex degradation system for the endogenous collagen makes the exogenous collagen highly biodegradable and low immunogenic. Recently, the attention on collagen degradation pathways has grown for the even more evident collagen critical role in tissue homeostasis [39]. Evidence about collagen and its degradation products could also be helpful in promoting the restoration of tissue structure and function [62].

## 4. Historical Overview on Collagen-Based Injectable Formulations

The history of biomaterials used as soft tissues filler dates from before the 19th century. The first injectable filler, which was autologous fat, was used in 1893 for forearm scar filling [63]. Since then, several materials have been used for the development of injectable formulations. Some of them were abandoned because of the development of medium-severe adverse reactions (e.g., paraffin: embolization, granuloma formation, migration; silicone: granuloma formation; teflon: inflammatory reaction) [15,64]. Among them, autologous fat is still used as filler for its biocompatibility, availability and low cost. However, its long-term variable and unpredictable results limited its employment [10,15].

A strong turning point happened in 1981 with the development and Food and Drug Administration (FDA) approval of the first collagen filler, Zyderm^®^ (Inamed Corporation, Santa Barbara, CA, USA). A new aesthetic procedure category of injectable treatments known as “fillers” was created. This paved the way for research into and development of biomaterials-based injectable formulations. However, the risk of immunogenic and hypersensitivity reactions soon decreased the popularity of animal-derived collagen fillers [64]. Moreover, the fear that the protein extracted from some animal tissues can be a vector for prion infections precluded their use. However, it should be taken into account that the first registered adverse events were not only related to material properties but also to the implantation methods. Proper patient selection and optimal methods of treatment delivery are crucial factors for therapeutic success and patient satisfaction [65]. Unfortunately, due to this, in the 1990s many collagen injection therapies failed because of the lack of data. Thereafter, surgeons were even more reluctant to perform collagen injections because they were commonly considered as ineffective therapies.

Thus, despite the growth of research interest in new fillers development, Zyderm^®^ remained the only FDA approved injectable formulation until 2003 when the first hyaluronic acid based dermal filler, Restylane^®^ (Galderma, Fort Worth, TX, USA, www.galderma.com, accessed on 14 February 2023), was approved. Since 2003, there has been an exponential increase in the number of FDA approved fillers. Indeed, both permanent (e.g., poly(methyl methacrylate), polyacrylamide, polyvinylpyrrolidone) and resorbable (e.g., collagen, hyaluronic acid, calcium hydroxyl apatite, poly(L-lactic acid) materials-based fillers were developed and clinically approved. Although synthetic compounds gained popularity as soft-tissue augmentation for their cost-effectiveness, mass production, limited immunogenicity and long-term effects, they also raised concerns over their long-term safety due to the growing data on long-term side effects or adverse events such as tissue necrosis, infection, granulomas, chronic inflammatory reaction [6,7,8,9,10,11,12].

In this context, resorbable fillers caught on even more for their relative safety in terms of local immunological reactions and ability to actively restore soft tissues volume. Indeed, the advantages offered by the use of minimally invasive therapies and the spread of the idea of regenerating damaged tissues pushed towards the development of temporary injectable hydrogels with specific properties. In particular, as argued by Cho et al., injectable bioactive formulations should: (i) be biocompatible without toxicity or immunogenic phenomenon after degradation; (ii) have mechanical properties compliant with the targeted tissue; (iii) be able to keep drugs and cells in the injected area; (iv) have adequate permeability, pore size and interconnectivity for mass transport and cell colonization; (v) be cost-effective; (vi) be easily handled; (vii) be biodegradable, allowing replacement by the newly formed functional tissue [66]. Indeed, an ideal injectable formulation should form a natural open pore 3D scaffold that should allow cell migration, and slowly break down stimulating growth factors and cytokines to promote neocollagenesis, elastic fiber production, neovascularization, and the wound healing response/repair [67]. Thus, ideal injectables should not only provide immediate and stable results, but also recreate natural-like extracellular matrix (ECM) for bio-dermal restoration and a long-lasting effect. However, one of the main disadvantages of resorbable filler is their short half-life. An inadequate reabsorption rate may not be sufficient to support the regenerative processes and therefore may lead to form loss. Thus, resorbable fillers-based approaches may require multiple applications to maintain their effect. 

For this reason, in the last two decades, type I collagen-based products and derivates (i.e., hydrolysates, gelatin, peptides) came back into vogue because of the spreading idea of developing multifunctional fillers able to fill soft tissue defects and restore deficient tissue physiological functions [4,12,14,32,66,68,69]. The use of heterologous collagen as a medical product spread also as results of the development of both accurate extraction processes and effective sterilization procedures that improved their safety profile. Indeed, advances in purification processes allowed creation of collagen preparations with minimum immunogenicity and infection risks, with high purity levels [19,25]. Moreover, with the definition of adequate implantation protocols, collagen-based injectable therapies were re-evaluated as a minimally invasive and effective strategies for the treatment of different types of diseases. Therefore, on account of collagen’s intrinsic structural and non-structural properties due to which it is historically considered as the “gold standard” material for the development of health-care related products, collagen-based injectable formulations have proved to be a promising strategy in many applicative areas. Despite the well-known effectiveness of collagen in tissue regeneration, the recent discovery of new ECM homeostasis molecular mechanisms raised again the interest in the mechanism of action of collagen. Indeed, lately it has been discovered that type I collagen operates a traction on the type VI collagen fibrils, which forms a network of fibrils in the immediate vicinity of the cell membranes [70]. The mechanical stress that results on the cells stimulates the production of new ECM (mechano-transduction process).

## 5. Collagen-Based Injectable Formulations

More than 60 kinds of collagen-based fillers are available on the market, according to the end-use and they have routinely been introduced in many clinical settings (Table 2). The most common collagen extraction sources for the manufacture of collagen based injectable formulations are bovine, swine, porcine, equine and human derived, whose advantages and disadvantages are described in depth elsewhere [19,20,25]. Bovine collagen is one of the most commonly used fillers for effectively reducing wrinkles and other facial imperfections. More famous branded bovine-based collagen fillers are Zyderm^®^, Zyplast^®^, Contigen^®^ (Allergan Inc., Dublin, Ireland), Artefill^®^ (Suneva Medical, San Diego, CA, USA), and Artecoll^®^ (Canderm Pharma Inc., Saint-Laurent, QB, Canada). Others include CHondroGrid^®^ (Bioteck Spa, Arcugnano, Italy), Integra Flowable Wound Matrix^®^ (Integra LifeScience Corp., Princeton, NJ, USA), Resoplast^®^ (Rofil Medical International, Breda, The Netherlands), Atelocell^®^ (KOKEN Co., Ltd., Bunkyo-ku, Tokyo, Japan). However, bovine collagen is known to be exposed to zoonosis (e.g., the foot and mouth disease and the group of the bovine spongiform encephalopathies, among which the most dangerous for humans is the transmissible spongiform encephalopathy) and to trigger allergies (about 2–4% of population) [71,72,73]. In addition to the strict regulation to which all implantable products are subjected, two consecutive negative patient skin tests at 6 and 2 weeks are required before use [73,74]. This sensitivity has been considered generally acceptable for implants for human use and actually bovine collagen is principally used for the treatment of the integumental [6,75,76,77,78,79,80,81,82,83,84,85,86,87,88,89,90,91,92,93,94,95,96] (NCT01060943) and musculoskeletal apparatus [97,98,99,100,101,102,103,104,105,106,107,108,109,110,111,112] and to a minor extent for the gastrointestinal [113,114,115,116,117,118,119,120], urinary [65,121,122,123,124,125] and cardiovascular [126,127,128] systems. Recently, bovine collagen in fibrillar form has been employed as an organ protection system during thermal ablation of hepatic malignancies [129].

Porcine collagen is the second most used. There are several products derived from porcine collagen, including GUNA^®^ (GUNA, Milan, Italy) products, CartiRegen^®^ (Joint Biomaterials S.r.l., Mestre, Italy), COLTRIX CartiRegen^®^ and TendoRegen^®^ (Ubiosis, Gyeonggi-do, Republic of Korea), CartiFill^®^, CartiZol^®^, RegenSeal^®^ and TheraFill^®^ (Sewon Cellontech Co., Ltd., Seoul, Republic of Korea), Dermicol-P35 (Evolence, Ortho Dermatologics, Skillman, NJ, USA), Fibroquel^®^ (Aspid S.A. de C.V., Mexico City, Mexico), Fibrel^®^ (Mentor Corporation, Santa Barbara, CA, USA), Permacol^®^ (Tissue Science Labs., Aldershot, UK) and RPC Pure Collagen^®^ (EternoGen LLC, Columbia, MO, USA). Among them, Dermicol-P35^®^, was withdrawn from the market in 2009. Compared to other animal derived collagens, porcine collagen-based injections are said to be rather painful and may cause allergic reactions [17]. While bovine collagen is used for many purposes, porcine collagen is almost exclusively used for the treatment of diseases belonging to the musculoskeletal apparatus [130,131,132,133,134,135,136,137,138,139,140,141,142,143,144,145,146] (NCT02539030, NCT02519881, NCT02539095), followed by the integumental apparatus [67,76,86,87,147,148,149,150,151,152] (NCT03844529, NCT00891774, NCT00929071) and gastrointestinal apparatus [116,117,153,154,155,156,157,158,159]. Only recently porcine collagen potential has been explored for the treatment of facial nerve palsy [160] and for the treatment of COVID-19 due hyperinflammation [161,162] (NCT04517162). However, despite their wide use and effectiveness, bovine and porcine collagens suffer from cultural or religious concerns (bovine collagen: Sikh, Buddhism; porcine collagen: Jewish, Islamic faiths), which restricted their applicative potential [18,19].

Equine collagen is the third most used collagen. It is free from the risks of triggering immune reaction and of zoonosis transmission, as reported elsewhere [19]. This kind of collagen is less used than bovine and porcine derived collagen for the manufacture of injectable formulations because of its naturally high hierarchical organization that made it more compliant for other applications (i.e., sponges, thin substrates). Thus, less injectable products from horse collagen are available but recently discovered advantages deriving from its use [19] are driving the development of new equine collagen-based products. Among them, Nithya^®^, Linerase^®^ (Euroresearch, Milan, Italy), Salvecoll-E^®^ (Nearmedic Italy S.r.l., Como, Italy), Biocollagen^®^ and ActivaBone^®^ (Bioteck Spa, Arcugnano, Italy) are commercially available and are mainly used for the treatment of diseases belonging to the integumental [163,164], urogenital [165] and gastrointestinal [160] apparatus. Its potential has also been recently explored for the treatment of periodontal tissues, with encouraging outcomes [166,167]. 

Human collagen fillers were developed in the early 2000s and are principally used for the integumental apparatus (e.g., facial soft tissues augmentation, wrinkles, scars, fat atrophy, diffuse depressions, paralyzed lips and tongues, nasolabial folds, and others) [6,168,169,170,171,172] and have been investigated for diseases of the gastrointestinal apparatus (e. g., vocal folds) [118,173,174,175]. In particular, there are three kinds of human collagen based injectables: autologous reconstituted collagen formulation (Autologen^®^ and Dermologen^®^, Collagenesis, Inc., Beverly, MA, USA) [173], autologous collagen formulations from in vitro cultured cells (Isolagen therapy^®^ from Fibrocell Science, Exton, Pennsylvania, USA,; Cosmoplast^®^ and Cosmoderm^®^ from Inamed Corporation, Santa Barbara, CA, USA)(NCT00655356)[6,169], and reconstituted collagen formulation from deceased humans (Fascian^®^ from Fascia Biosystem, Beverly Hills, CA, USA; Dermalogen^®^ and Cymetra^®^ from Life Cell Corp., Branchburg, NJ, USA) [6,118,168,170,171,172,173]. Autologous reconstituted collagen formulations are produced from collagen harvested from patients’ skin small biopsy, harvested during an earlier procedure, and liquefied for future re-injection. Two square inches of donor material are required to formulate a 1-mL syringe of injectable material, which can be stored for 6 months [176]. This procedure was developed by Collagenesis Inc. (Beverly, MA, USA) and is commercially known as Autologen^®^. As previously noted, human collagen fillers can also be derived from in vitro cultured autologous cells. In particular, skin cells from behind the human ear could be harvested, cloned, and derived collagen could be then harvested, liquefied, and injected. This procedure was developed by Fibrocell Science Inc. (Exton, PA, USA) and is commercially known as azfibrocel-T (formerly Isolagen Therapy^®^). Being autologous collagen, these kinds of formulations are allergy free, making them an excellent alternative to animal-derived treatments. Apart from general mild disorders (bruising 5%, erythema 15%, hemorrhage 10%, with numbers comparable to placebo groups), this kind of human derived formulation does not trigger serious adverse events (NCT00655356). Human collagen fillers could also be prepared from deceased human donors, with the main advantages of extensive raw material availability and the reduced preparation time compared to both autologous reconstituted collagen formulation and autologous collagen formulations from patients’ own in vitro cultured cells. Injectables from human donors (Dermalogen^®^) were firstly developed by Life Cell Corporation (Branchburg, NJ, USA). Because Dermalogen^®^ originates from humans, also the deceased human-derived collagen-based injectables do not need an allergy test. Although human-derived collagens proved to be a good alternative, they have some disadvantages such as long preparation times, non-availability of sufficient donor tissue and high management costs (i.e., harvesting, donor tissue availability, isolation, manufacturing, need for highly specialized teams and instruments, refrigerated and limited storage, shipping) [7,176,177]. Moreover, while no efficacy differences emerged between the use of autologous collagen and animal-derived collagen, a 2–3 folds greater injection of cadaveric collagen is needed for similar augmentation results to those achieved with bovine collagen [176]. Thee mentioned drawbacks, together with the insubstantial difference in terms of efficacy compared to animal-derived collagen-based injectables, led to the progressive abandonment of human collagen for large scale applications and its exclusive use for patients with hypersensitivity to animal derived collagens.

In the last decade, new solutions were offered by recombinant collagens. Indeed, two injectable fillers, consisting of collagen, hyaluronic acid and carboxymethylcellulose, are now commercially available. In particular, Fillagen^®^ (Monodermà, Milan, Italy), made with recombinant polypeptide of collagen α1-chain from silkworm [178], and Karisma^®^ (Taumed, Rome, Italy), made with unspecified recombinant collagen were proposed. More recently a photocurable collagen-based regenerative dermal and soft tissues filler was developed by CollPlant Biotechnologies Ltd (Rehovot, Israel, www.collplant.com, accessed on 14 February 2023), comprising a recombinant type I collagen from tobacco plant (not currently commercially available).

**Table 2:** Summary of clinically available type I collagen-based injectable formulations.

Source	Manufacturer	Product	Additives	Applications	Ref.
Equine	Euroresearch S.r.l. (Milan, Italy) www.euroresearch.it, accessed on 14 February 2023	Nithya	–	Integumental	[163]
		Linerase	–	Integumental	[164,165,166,167,179]
	Nearmedic Italy S.r.l. (Como, Italy) www.salvecoll.com, accessed on 14 February 2023	Salvecoll-E	–	Integumental	[60]
	Bioteck Spa (Arcugnano, Italy) www.bioteck.com, accessed on 14 February 2023	Biocollagen gel	Type III collagen, bone spongy powder	Musculoskeletal	–
		Biocollagen crunch	Type III collagen, bone powder, bone spongy chips	Musculoskeletal	–
		ActivaBone CLX gel	Bone powder, exur, Vitamin C	Musculoskeletal	–
		ActivaBone Injectable Paste	Demineralized bone matrix, bone powder, exur, Vitamin C	Musculoskeletal	–
		ActivaBone modulable paste	Demineralized bone matrix, bone powder, bone cortical and spongy granules, exur, Vitamin C	Musculoskeletal	–
		ActivaBone Crunch	Demineralized bone matrix, bone powder, cortical and spongy chips, exur, Vitamin C	Musculoskeletal	–
Bovine	Bioteck Spa (Arcugnano, Italy) www.bioteck.com, accessed on 14 February 2023	CHondroGrid	–	Musculoskeletal	[112]
	Integra LifeScience Corp. (Princeton, NJ, USA) www.integralife.com, accessed on 14 February 2023	Integra Flowable Wound Matrix	Glycosaminoglycans	Integumental	[88]
		Helitene	–	Soft tissues	[129]
	Rofil Medical International (Breda, The Netherlands)	Resoplast	Lidocaine hydrochloride	Integumental	–
	Suneva Medical (San Diego, CA, USA) www.sunevamedical.com, accessed on 14 February 2023	ArteFill	Polymethylmethacrylate, lidocaine	Integumental	[75,77,78,79,80,81,82,83,84,85]
	Datascope Corp., (Montvale, NJ, USA)	VasoSeal	–	Cardiovascular	[128]
	BioMimetic Therapeutics, LLC (Franklin, TN, USA) www.biomimetics.com, accessed on 14 February 2023	Augment	β-tricalcium phosphate, recombinant human platelet-derived growth factor-BB	Musculoskeletal	[97,99,100,101,102,103,104,105,106,107,108,109,110,111]
	KOKEN Co., Ltd. (Bunkyo-ku, Tokyo, Japan) www.kokenmpc.co.jp, accessed on 14 February 2023	Atelocell	Type III collagen	Integumental, gastrointestinal	[86,87,113,114], NCT01060943
	B. Braun (Crissier, Switzerland) www.bbraun.com, accessed on 14 February 2023	Gelofusine	–	Cardiovascular	[126,127]
	Allergan, Inc. (Dublin, Ireland) www.abbvie.it, accessed on 14 February 2023	Zyplast	Glutaraldehyde	Integumental	[6,76,83,89,90,91,92,95,96,98,116,117,119,180]
		Zyderm	–	Integumental	[6,83,89,90,93,94,118,120,180]
		Contigen	glutaraldehyde	Gastrointestinal and genitourinary	[115,121,122,123,124,125]
Swine	GUNA (Milan, Italy) www.guna.com, accessed on 14 February 2023	Dental Skin BioRegulation	Vitamin C, magnesium gluconate, pyridozine chlorhydrate, riboflavin, thiamine chlorhydrate	Skin	[181]
		Dental ATM BioRegulation	Hypericum	Musculoskeletal	[130]
		MD-HIP	Calcium phosphate	Musculoskeletal	[131]
		MD-ISCHIAL	Rhododendron	Musculoskeletal	[132]
		MD-KNEE	Arnica	Musculoskeletal	[133,143,144]
		MD-LUMBAR	Hamemelis	Musculoskeletal	[132,134,135]
		MD-NECK	Silicio	Musculoskeletal	–
		MD-SHOULDERS	Iris	Musculoskeletal	[145,146]
		MD-SMALL JOINTS	Viola	Musculoskeletal	–
		MD-THORACIC	Cimifuga	Musculoskeletal	–
		MD-MATRIX	Citric acid, nicotinamide	Soft tissues	[135,136,160]
		MD-MUSCLE	Hypericum	Musculoskeletal	[130,132,133,134,135,136,137,146,160]
		MD-POLY	Drosera	Musculoskeletal	–
		MD-NEURAL	Citrullus	Musculoskeletal	[132,134,160]
		MD-TISSUE	Ascorbic acid, magnesium gluconate, pyridoxine chlorhydrate, riboflavin, thiamine chlorhydrate	Soft tissues	–
	Joint Biomaterials S.r.l. (Mestre, Italy) www.joint-biomateriali.it, accessed on 14 February 2023	CartiRegen	Fibrin glue	Musculoskeletal	–
	Ubiosis (Gyeonggi-do, Republic of Korea) www.ubiosis.com, accessed on 14 February 2023	COLTRIX CartiRegen	–	Musculoskeletal	–
		COLTRIX TendoRegen	–	Musculoskeletal	–
	Sewon Cellontech Co., Ltd. (Seoul, Republic of Korea) www.swcell.com, accessed on 14 February 2023	CartiFill	Glucose, CaCl, amino acids, vitamin B, fibrin glue	Musculoskeletal	[138,139], NCT02539030, NCT02519881
		CartiZol	Glucose, CaCl, amino acids, vitamin B	Musculoskeletal	[140], NCT02539095
		RegenSeal	–	Musculoskeletal	[141]
		TheraFill	–	Integumental	[86,87]
	Sunmax Biotechnology Co., Ltd. (Tainan, Taiwan) www.sunmaxbiotech.com, accessed on 14 February 2023	Facial Gain	Lidocaine	Integumental	NCT03844529
		Collagen Implant I	–	Integumental	–
	Evolence (Skillman, NJ, USA)	Dermicol-P35	Ribose	Integumental	[2,147,148,149], NCT00929071, NCT00891774
	Mentor Corp. (Santa Barbara, CA, USA)	Fibrel	–	Integumental	[150,151]
	Tissue Science Labs. (Aldershot, UK)	Permacol	–	Gastrointestinal	[153,154,155,156,157,158,159]
	EternoGen, LLC (Columbia, MO, USA)	RPC Pure Collagen	Ethylenediamine tetraacetic acid	Integumental	[67]
	Aspid S.A. de C.V. (Mexico City, Mexico) www.aspidpharma.com, accessed on 14 February 2023	Fibroquel	Polyvinylpyrrolidone	Musculoskeletal	[161,162], NCT04517162
	ColBar LifeScience Ltd. (Tel Aviv, Israel) www.ortho-dermatologics.com, accessed on 14 February 2023	Evolence	Ribose	Integumental	[147,152]
Human	Fascia Biosystem (Beverly Hills, CA, USA)	Fascian	Lidocain	Integumental	[6,168,171]
	Fibrocell Science (Exton, PA, USA) www.fibrocell.com, accessed on 14 February 2023	Isolagen therapy	–	Integumental	NCT00655356
	Inamed Corporation (Santa Barbara, CA, USA) www.inamed-cro.com, accessed on 14 February 2023	Cosmoplast	Glutaraldehyde, lidocaine hydrochloride	Integumental	[6,169]
		Cosmoderm	lidocaine hydrochloride	Integumental	[6,169]
	Life Cell Corp. (Branchburg, NJ, USA)	Dermalogen	Type and VI collagen, elastin, fibronectin, chondroitin sulfate, and other proteoglycans	Integumental	[170]
		Cymetra	Elascin, glycosaminoglycans, Lidocaine hydrochloride	Integumental	[6,118,172,173,174,175]
	Collagenesis, Inc., (Beverly, MA, USA)	Autologen	Elastin, fibronectin, glycosaminoglycans	Integumental	–
		Dermologen	-	Integumental	[173]
Plant	Vesco Pharmaceutical Co. Ltd. (Bangkok, Thailand) www.vescopharma.com, accessed on 14 February 2023	Collagen C 1000	Vitamin C	Integumental	–
Silkworm	Monodermà (Milan, Italy) www.monoderma.com	Fillagen	Hyaluronic acid, carboxymethylcellulose	Integumental	[178]
n. d.	Taumed (Rome, Italy) www.taumed.it, accessed on 14 February 2023	Karisma	Hyaluronic acid, carboxymethylcellulose	Integumental	–
n. d.	LABO International S.r.l. (Padova, Italy) www.labosuisse.com, accessed on 14 February 2023	Fillerina con 3D collagen	Hyaluronic acid	Integumental	–
n. d.	Hebey Mepha Pharm Group Co., Ltd. (Shandong, Hebei, China) www.mephacn.com, accessed on 14 February 2023	Collagen Plus	–	Integumental	–
n. d.	Pierre Mulot Laboratories (Paris, France)	Neutroskin	Vitamin C	Integumental	–
n. d.	Elements Pharmaceuticals (Shijiazhuang, Hebei, China) www.elementspharma.com, accessed on 14 February 2023	Ele-collagen	Vitamin C, Vitamin B6	Integumental	–
n. d.	Globus Medical (Audubon, PA, USA) www.globusmedical.com, accessed on 14 February 2023	Kinex Bioactive gel	Bioglass, hyaluronic acid	Musculoskeletal	–

## 6. Clinical Efficacy of Collagen-Based Injectable Implants

**Figure 2:**
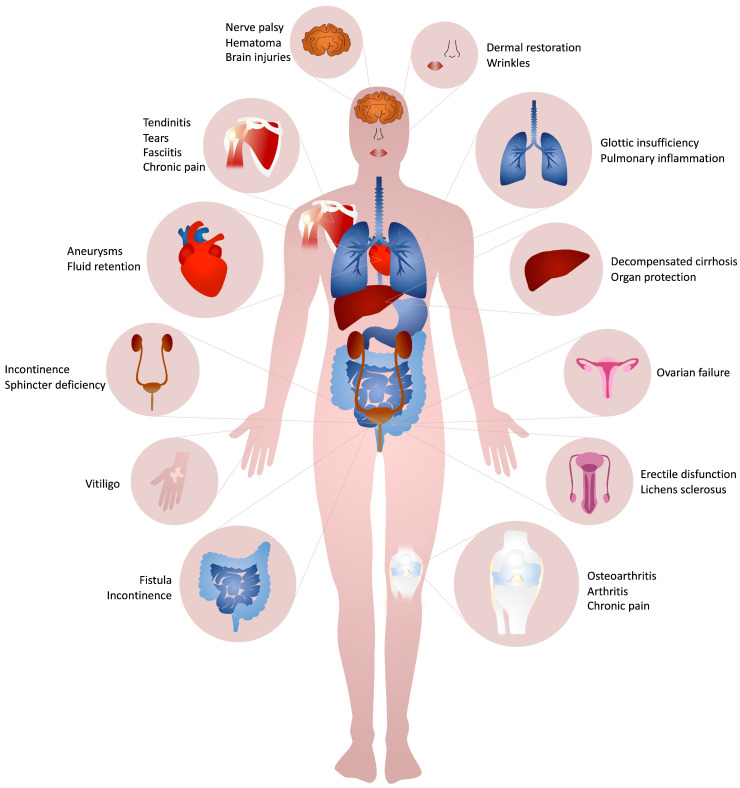
Main collagen-based injectables applications for the treatment of several kind of diseases belonging to the integumental, musculoskeletal, urogenital, gastro-intestinal apparatus, besides for non-standard clinical applications.

Collagen-based formulations are mainly used for the treatment of several kind of diseases belonging mainly to the musculoskeletal (i.e., hip or knee osteoarthritis [112,131,133,140,142,144,182], sprained knee pain [143], injured cartilage [138,141], piriformis syndrome [136], ankle and hindfoot arthritis [103] or fusion [100,106,107,108,109], lumbar spinal fusion [99], myofascial pain syndrome [130,137], chronic pain [132], acute lumbar spine pain [134], partial-thickness rotator cuff tears [141,146,183], plantar fasciitis [184], calcific supraspinatus tendinitis [145], pain [130,132,134,137]), urogenital (i.e., urinary incontinence [122,124,125,185,186,187,188,189], neurogenic urinary incontinence [190], lichens sclerosus [165], intrinsic sphincter deficiency [191,192,193], post-prostatectomy incontinence [65,123,194,195,196,197], retrograde ejaculation [198]), gastrointestinal (i.e., glottic insufficiency [113,114,116,118,119,173,199,200,201,202,203], rectal fistula [153,154,156,157], fecal incontinence [69,115,155,204]), and integumental (i.e., nasolabial folds [2,67,76,86,87,96,149,163,172,205,206,207,208], nasojugal folds [152], lip [2,77,95,148,169,172], cheek and temple area [172], glabellar groove [77], post-rhinoplasty dorsal irregularities [77,209], depressed acne scars [77,172,210] augmentation, post-burn hands malfunction [88] and vitiligo [164]) systems, as well as for non standard clinical applications (i.e., facial nerve rehabilitation after palsy [160,211], organ protection during thermal ablation [129], COVID-19 associated hyperinflammation [161,162] (NCT04517162), vitiligo [164], ovarian function after premature ovarian failure [212], the closure of artery aneurysms [128,213] and blood volume augmentation [127,214]) (Figure 2).

However, many manufacturers have chosen to not publish their findings but keep their data privately on file. Thus, no public clinical efficacy research results are available for many injectable solutions, meaning that the limited information available restricts the discussion on the efficacy and safety of collagen-based formulations for healthcare applications.

### 6.1. Integumental Apparatus

Type I collagen is the main component of skin (85–90% type I collagen, 10–15% type III collagen). Fibrillar collagen types I, III, and V self-assemble into larger collagen fibers that form the dermis 3D network [215]. 

To improve the appearance of aged skin many non-invasive (i.e., topical formulations, oral supplements), minimally invasive (i.e., dermal fillers) and surgical treatments (i.e., blepharoplasty) were developed. Although a multitude of topical treatments are available for the improvement of aged skin appearance, these procedures appeared to have minimal ability to remodel dermal ECM [215]. However, collagen supplements originating from various animal sources such as marine, bovine, and porcine were revealed to be able to partially improve skin integrity. Thus, injectables became more popular for their immediate effect. As previously noted, several biomaterials (i.e., collagen, hyaluronic acid, calcium hydroxyl apatite, carboxy methyl cellulose, poly (methyl methacrylate), poly(L-lactic acid) were employed for the development of skin filler, each of which has some advantages and drawbacks. 

Among them, collagen is the most promising for its low adverse effects rate and natural filling effect. The return to favor of collagen injectables for aesthetic medicine could be due to the acquired knowledge about chronological skin aging processes. Wrinkles formation is caused by collagen density decrease due to its turn-over slowing-down [215]. Its decreased synthesis and replacement rate causes matrix loss and thus skin collapse and loss of elasticity, which in turn leads to the appearance of wrinkles, folds, and facial contour changes, as masterfully described by Fisher et al. 2008 [215]. Due to this, several commercial collagen-based products are available and are used principally for facial contouring, such as for nasolabial folds [2,67,76,86,87,96,149,163,172,205,206,207,208], nasojugal folds [152], lip [2,77,95,148,169,172], cheek and temple area [172], glabellar groove [77], post-rhinoplasty dorsal irregularities [77,209], depressed acne scars [77,172,210], augmentation [96,172].

Usually, collagen injectable formulations for antiaging treatments are supplied in the form of dry powder to be resuspended in a suitable buffer (e.g., NaCl 0.9%) or in liquid form in ready-to-use syringes with a final collagen concentration of 30–35 mg/mL. A total of 2–5 mL is injected to reach the desired effect. In particular, a volume of about 0.9–3.0 mL [67,86,95,149,205,206,207,208] is injected in the first session, but because of collagen’s rapid degradation, 1–3 touch-up treatments [67,87,96,149,206] of about 0.8–2.1 mL [67,149,206] are usually performed usually after 1–2 week from the first treatment [67,76,86,87,96,149,208] and more rarely after 1 [206] or 6 months [207].

An improvement of the Crows’ feet severity, Facial Volume Loss Scale (FVLS), and Wrinkle Severity Rating Scale (WSRS) of about 1 point was registered almost always after 6–8 months [86,163]. Accordingly, the improvement of the Global Aesthetic Improvement Scale (GAIS), Merz Aesthetic Scale (MAS) after 12 weeks of at least 1 point was registered in another study [67]. The maximum WSRS improvement of about 0.5–1.0 point was usually reached after 3 months [96,205,206]. However, the WSRS and GAIS score were reduced by about 0.5–1.0 and 1.0–1.5 points respectively after 12–24 months, confirming the biodegradability of collagen fillers and therefore the need to resort to multiple injections to maintain the desired effect [87,96]. Indeed, based on overall Subject Global Evaluation scores, patients reported 96% aesthetic improvement at the week-3 follow-up visit, a value that decreased to 60% at month-3 and to 15% at month-13 post–last treatment visits [205]. However, collagen persistence has been successfully improved by about 2 points of the WSRS scale after 6 months and prolonged with the use of crosslinked collagen (Dermicol-P35) up to 1 year with 1 touch-up after 1 week without immediate or delayed positive hypersensitivity reactions [149,208]. Collagen filler injected volume or its animal extraction may not be the influencing factor of collagen efficacy or side effects. Several studies reported how injected volume did not differ significantly between porcine collagen formulation (i.e., 2.03–2.11 mL for nasolabial fold, 0.90 mL for lip) and bovine collagen formulation (i.e., 1.8–2.1 mL for nasolabial fold, 0.85 mL for lip) nor were statistically significant differences registered in WSRS and GAIS score improvement, patients’ satisfaction and adverse events [86,87,95]. Collagen fillers (Dermicol-P35, Artecoll^®^, Cymetra) demonstrated their effectiveness also in cases of depressed acne scars since they allowed to reach a high degree of correction, with no adverse events and high patient satisfaction level [77,172,210]. Although the acne scars were not completely removed, their appearance was greatly improved [210].

Collagen filler is generally considered safe. As shown in Table 3, fillers were well tolerated and there were no serious adverse reactions [67,87,96,163,216]. Indeed, serious adverse events that were not injection site related usually not occur [67]. However, all injection site reactions were mild to moderate in severity and resolved in 1–2 weeks without sequalae [2,86], except for some rare cases. Usually, 80% of participants had at least 1 injection site reaction after the initial injection [67]. Light/moderate bruises appear on the injection points that totally disappeared within 5–10 days [163]. Only one severe bruising was reported after 1-week follow-up and resolved after 4 weeks [67]. A case of mild induration after 4 weeks resolved in 6–12 weeks [67].

As previously mentioned, with the spreading idea of regenerating lost tissues rather than repairing them, collagen formulations started to be employed not only for aesthetic medicine but also for regenerative medicine. Only recently, collagen fillers started to be used for the treatment of other integumentary apparatus diseases such as the post-burn hands malfunction [88] and vitiligo [164]. Indeed, the potential of a collagen-glycosaminoglycans matrix (INTEGRA^TM^ Flowable Wound Matrix) has been investigated for post-burn hands treatment with the idea that its composition was supposed to have the potential to rebuild the lost or injured deep dermal structure and enable soft tissue augmentation [88]. The work of Hirche et al. was the first pilot study using percutaneous injectable collagen-glycosaminoglycans matrix for post-burn dermal augmentation safety and efficiency, and active range of motion (AROM), disabilities of the arm, shoulder and hand (DASH) score, Vancouver scar scale (VSS) score and scar quality improvement were registered after 6 months [88]. Despite the encouraging results, further studies on the formulation’s long-term efficacy on a higher number of patients are necessary in order to evaluate the possible quality of life and grip strength that appeared to not be changed after 6 observation months [88]. More recently, collagen injections were proposed also for unresolved diseases such as vitiligo. Although a variety of treatments for the re-pigmentation of vitiligo lesions are available (e.g., platelet-rich plasma injections, UV-phototherapy), none of them effectively promote complete and long-lasting re-pigmentation. Thus, the potential synergistic effect of intradermal collagen injections (Linerase^®^) in combination with UV-phototherapy was investigated and 70% re-pigmentation occurred after six sessions with mild-to-moderate pain and no adverse events [164]. Moreover, no relapses were reported after one year [164]. 

**Table 3:** Clinical trials details (i.e., participants, injections number, volume and administration time (weeks (w)) and adverse events recurrence on collagen based injectable formulations for skin rejuvenation from 2000 to 2022.

Collagen Source		Equine	Swine																Bovine							Human				
Product Name		Nithya	Dermicol-P35										Permacol	Therafill			RPC Pure-Collagen	Sunmax FacialGain	Zyplast				Artecoll	Koken		CosmoDerm		CosmoPlast		Isolagen
Injection specification	Number (n)	1	2	2	1	1–2	1–2	2	2	1	1	1	1	n. d.	1	1	2	1	1	3	1–3	1	1	1	1	1	1	1	1–4	3
	Volume (mL)	2–5	2.2	2	n. d.	n. d.	0.6	1	1	1	n. d.	n. d.	1.5–3.0	1	2	2	2.56	n. d.	1.5–3.0	2	1.6	1	n. d.	1.8	2	1	n. d.	n. d.	1	n. d.
	Amount (mg)	80	77	70	n. d.	n. d.	21–42	70	70	35	n. d.	n. d.	52–105	30	60	60	89.6	n. d.	53–105	70–210	56–168	35	n. d.	54	70	35	n. d.	n. d.	n. d.	n. d.
	Interval (inj./weeks)	1	0.5/w	0.5/w	n. d.	0.5/w	0.25/w	0.04/w	0.25/w	n. d.	n. d.	n. d.	n. d.	0.5/w	n. d.	n. d.	1/w	n. d.	n. d.	0.8/w	0.5/w	n. d.	n. d.	n. d.	n. d.	n. d.	n. d.	n. d.	0.3/w	0.6/w
Observation time (weeks)		24	48		48	24	32	n. d.	8	1	1	240	48	12	24	48	12	52	48	24	24	12	136	24	48	6	24	6	100	29
Participants (n)		72	148	24	20	172	12	1	1	10	30	1	19	73	57	57	30	n. d.	18	439	138	118	153	57	57	3	n. d.	1	117	110
Adverse events	Severe	0	0	149	0	0	0	0	0	0	0	1	0	n. d.	0	0	1	n. d.	0	0	0	0	0	0	0	0	n. d.	0	0	0
	Non severe	n. d.	5	0	16	154	0	0	0	0	38	0	18	n. d.	12	1	0	n. d.	1	n. d.	124	109	n. d.	18	5	1	n. d.	1	42	32
	Allergic reaction			n. d.									1																2	
	Pain										9				1						58	58							4	
	Infection					12							9						1											8
	Papule/erythema	v		80		17					14				7		24			391		75		8	1	1		1	4/31	14
	Nodule/lumpiness		1		16															369		15	52		1				7	
	Bruise	v		27							1						1			241	66	51	20						1	5
	Edema/swelling			42							22				2	1	24			378	101	61		6	3			1	5	
	Hemorrhage																												3	10
	Itching																24			##		24		1				1	9	
	Induration/tenderness		3	36											1					391	88	14		2					1	
	Discoloration		1			12									1					149		31							2	
	References	[163]	[149]	[208]	[148]	NCT00891774	[209]	[207]	[210]	NCT00929071	[2], NCT00911872	[147]	[95]	NCT01060943	[86]	[87]	[67]	NCT03844529	[95]	[96]	[76]	NCT00876265	[77]	[86]	[87]	[217]	NCT01212809	[217]	[205,206], NCT00444210, NCT00444353	NCT00655356

### 6.2. Musculoskeletal Apparatus

Aging leads not only to skin texture loss but also to a progressive and gradual reduction of all human capabilities. The loss of muscle or osteochondral mass with advancing age is the major public health problem for the elderly population. Thus, musculoskeletal apparatus-related medical treatments and costs increase with population age (numbers over 50 years). Among invasive and non-invasive currently available treatments, collagen injections are revealed to be quite effective for the treatment of several musculoskeletal diseases such as hip [131] or knee osteoarthritis [112,133,140,142,144,182], sprained knee pain [143], injured cartilage [138,141], piriformis syndrome [134], ankle and hindfoot arthritis [103] or fusion [100,106,107,108,109], lumbar spinal fusion [99], myofascial pain syndrome [130,137], chronic pain [132], acute lumbar spine pain [134] and in partial-thickness rotator cuff tears [141,144,183], plantar fasciitis [184], and calcific supraspinatus tendinitis [145] and pain [130,132,134,137].

Osteoarthritis is an inflammatory degenerative disease characterized by the progressive damage of articular cartilage and underlying bone that predominantly affects hip and knee [218]. Interleukin (IL)-1β, tumor necrosis factor (TNF)-α, and IL-6 seem to be the main proinflammatory cytokines involved in the pathophysiology of osteoarthritis, even though others, including IL-15, IL-18, IL-21, leukemia inhibitory factor (LIF), and chemokines are implicated [182,219]. The expression of these inflammation mediators in turn activates the cartilage-degrading enzymes, that are matrix metalloproteinases (MMPs) and A disintegrin metalloproteinase with thrombospondin motifs (ADAMTS) [112,219], that progressively degrade the ECM, including collagen. From this observation, several studies were performed to prove the hypothesis that an exogenous administration of collagen may be beneficial to osteoarthritis damaged cartilage and bone.

Indeed, a total of 12 mL of collagen and polyvinylpyrrolidone based formulation (Fibroquel^®^) affected the values of the Lequesne index (LKI) by −51%, Western Ontario and McMaster University Index (WOMAC) pain subscale by –51%, WOMAC stiffness subscale by −49%, WOMAC disability subscale by −42%, and the use of analgesics by –83% after 6 months [182]. Moreover, pro-inflammatory cytokine expression was lowered in patients under collagen treatment compared with placebo [192]. Injections of collagen, arnica and hypericum (MD-Knee^®^ and MD-Muscle^®^) brought a significant reduction of Visual Analogue Scale (VAS) pain at rest with a decrease of the average score for pain during movement of more than two-fold after 12 weeks [133]. Similar results were obtained by Martin et al. who found a LKI and VAS significant improvement 6 months after five weekly injections (a total of 20 mL) of MD-Knee^®^ [144]. More recently, analogous clinical outcomes were obtained with pure collagen formulations, with a reduced number of injections. Indeed, three injections of a hydrolyzed collagen suspension (a total of 6 mL of CHondroGrid^®^) significantly reduced VAS, LKI, and WOMAC scores [112], by up to about 50% [142]. 

Because of the avascular, aneural, and immunoprivileged nature of hyaline cartilage, the regenerative potential of cartilage after injury is limited. In this circumstance, collagen injections revealed to be a promising modality for single-stage cartilage repair: collagen augmented chondrogenesis by 50% filling of the microfractures with CartiFill^®^. This showed a superior VAS improvement rate analysis and a superior filling rate in the cartilage tissues as well as integration with the surrounding tissues 24 months postoperatively compared with that achieved only with microfracture [139].

Peri-articular collagen injections (MD-Knee^®^ and MD-Matrix^®^) twice/week for 3 consecutive weeks revealed to be effective also for the treatment of sprained knee pain, with a rapid recovery and an excellent control of breakthrough pain without the use of anti-inflammatory drugs [143]. 

Thus, all clinical outcomes confirmed the benefits in collagen use and allowed to define intra-articular collagen injection as inflammation down-modulator and cartilage regenerator ‘biodrug’ [182]. Collagen can effectively promote repair processes of the cartilage matrix, interrupting the degenerative process and articular damage, which causes inflammation and pain [144]. The administration of type I collagen after arthroscopic lavage is safe and effective and induced systemic inflammation downregulation [182]. Adverse events are rare, most frequently including site pain that lasts at most 24 h [131,182]. No aseptic acute arthritis, infections after injection or any other complication have ever been registered [112,131,182]. Taking into account that osteoarthritis is the most common form of musculoskeletal disorder with a prevalence of 23% of over 40 people and an annual incidence of 203 per 10,000 person/year [220], it is easy to understand how it has high economic costs and a devastating impact on patient quality of life. The above-mentioned recent studies showed how the benefits associated with the use of collagen make it a very promising non-invasive solution that has begun to find its place among conventional therapies (i.e., corticosteroids, polynucleotides, platelet-rich plasma, hyaluronic acid intra-articular injections). Although today collagen injections are still less popular than hyaluronan, they exert a similar clinical effect, besides being equally well tolerated both locally and at a systemic level, confirming the material non-inferiority [144]. The reduced cost of collagen-based formulations compared to hyaluronic acid-based formulations could bring to the attainment of the intra-articular therapy to a broad range of the population, resulting in the reduction of social cost due to working days lost and caregivers’ time off work [144].

Osteochondral disorders are followed by less common but equally disabling muscular and tendon pain. Inflammatory or degenerative process, fracture, radicular syndrome, or nonspecific syndrome are causes of chronic musculoskeletal pain, which is the most common health complaint, with significant social and economic consequences [132,134]. The incidence of musculoskeletal pain increases with age and strongly affects the quality of life of a growing number of affected people [137]. Current medical procedures include conservative methods (i.e., rehabilitation, medications), minimally invasive interventions (e.g., acupuncture) or surgical treatment. However, the huge risk of gastrotoxicity, hepatotoxicity, cardiotoxicity and nephrotoxicity, after long-term and/or high doses of common nonsteroidal anti-inflammatory drugs, pushed researchers toward the investigation of safer options [134]. In this circumstance, the subcutaneous/intramuscular administration of collagen containing products (MD-Lumbar MD^®^, MD-Muscle^®^, MD-Neural^®^) represented a new concept in the treatment of pain, that is based on the strengthening the collagen matrix underlying the musculoskeletal system structures and on the analgesic effects of these products. Although few published data are available, it is clear that collagen-based injections represent a safer treatment option with no adverse events, 54–60% pain relief [130,132], good tolerability [134], and comparable or better efficacy with the standard treatments [130,134]. 

Only recently the efficacy of collagen injections (RegenSeal^®^, MD-Shoulder^®^, MD-Muscle^®^) for the treatment of tendon tear have been clinically investigated [141,183,184]. The first prospective, randomized clinical trial has been conducted by Kim et al. in 2020 and reported rotator cuff functional outcomes improvement and a decreased tear size in 37% of patients with a single collagen injection (0.5–1.0 mL) [141]. A case study confirmed how multiple intratendinous weekly injections of 2 mL of collagen are able to reduce the partial-thickness tear in three months and to completely heal tendon tear in 18 months, which in addition appeared quite regular and isoechoic [183]. Collagen injections were thus found to be effective to decrease tear size (50–77% complete recovery), pain, increase functional shoulder score and delay tear progression in partial-thickness rotator cuff tears [141,145,146,183]. The precise mechanism of tendon healing after injection of collagen is still unknown. However, two in vivo studies on rabbits proved that injections of collagen in the tissue during the ECM remodeling phase led to better tendon healing and earlier progression to the remodeling phase [141,221,222]. Both histological and biomechanical studies of type I collagen implants facilitated continuity of injured tendons, decreased peritendinous adhesion, and improved muscle activity in Achilles tendons of rabbits [221,222]. Despite their low efficacy rate and their limited use, collagen injections would be more advantageous than traditional surgery for their cost-effectiveness, easy performance and less time-consuming nature. 

### 6.3. Urogenital System

Collagen injections have been revealed to be a minimally invasive and quite effective solution for specific urogenital system diseases such as stress urinary incontinence [122,124,125,185,186,187,188,189], neurogenic urinary incontinence [190], lichens sclerosus [165], intrinsic sphincter deficiency [191,192,193], post-prostatectomy incontinence [65,123,194,195,196,197], retrograde ejaculation [198] and ovarian function after premature ovarian failure [212].

Stress urinary incontinence affects 10–30% of women above 50 years of age [185]. To solve this common issue, in addition to surgical practices (i.e., retropubic bladder neck suspension or slings), biomaterials injections (i.e., teflon, fat, silicone, collagen) have been performed to increase urethral strength and avoid urinary leak. Among them, collagen (Contigen^®^, Linerase^®^) has remained the most promising. In a study of Martins et al., either cure or improvement was achieved in 86% of women, with a registered leak pressure increase and reduction in urinary protector use and urine leakage volume [185]. In another study, 48% were totally dry and 31% were socially continent after 2 months [187]. However, because of collagen absorption, stress urinary incontinence recurrence occurred in 41% of patients who achieved continence after 7–8 months [187]. Collagen reportedly degraded completely within 10–19 weeks, although magnetic resonance imaging of the urethra showed the persistence of the implant for as long as 22 months after injection [196]. Thus, repeated injections (2–5) may be necessary [187,188,190]. Hence, reinjections were performed, with a 42% regain of continence, giving a long-term success rate of 58–60% [187]. Totally favorable results, including improvement (40%) and cure (30%), were also recorded for up to 4 years [124]. However, it should be mentioned that elderly patients should be counseled that approximately 40% will experience recurrent leakage, which may not resolve with reinjection [187]. Conversely, Gorton et al. reported the absence of correlation between long-term success and the number of previous operations, body mass index, age, number or total volume of collagen injections [125].

Men’s post-prostatectomy incontinence incidence ranges from 2% to 87% [123]. The most commonly performed surgical procedures include the insertion of an artificial urinary sphincter or of injectable bulking agents. In this case, collagen (Contigen^®^) is the most commonly used and several works reported how 3–4 collagen injections led 8–20% of patients to dryness and 38–39% to significant improvement [123,194]. Treatment was found to be pad related. The highest success rate was reached in patients that fewer than 6 pads per day (72%) a value that lowers up to 29% for patients using more than 6 pads per day [123]. Moreover, in cases of radiation therapy or bladder neck incision after a radical prostatectomy, the success rate is even lower [123]. The success rate of collagen injection strongly decreased in the treatment of urinary incontinence in children with neurogenic bladder dysfunction secondary to myelomeningocele. In this case, only 15% improved and 5% were completely dry [190]. Additionally, the initial improvement in the first 2 months after injection deteriorated thereafter in 80% of children [190]. The first severe case was registered in 2006, when three years after a single sub ureteral collagen injection for the treatment of bilateral vesicoureteral reflux in a 1 year of age girl, hydronephrosis with ureteral stenosis with a knotty sclerosis and a histiocytic and granulomatous reaction occurred and required ureteral reimplantation [223]. Despite the widespread and long-term application of collagen for the treatment of stress urinary incontinence, treatment-related morbidity was minimal. Urinary tract infections occurred in 6% to 25% of cases while transient hematuria and hypersensitivity were occasionally reported [124,125]. No implant migration, nor seroconversion to antibodies that cross-reacted with human collagen, nor symptoms were even registered [123]. However, patients who have required a penile clamp and experienced continuous leakage or those who have undergone transurethral incision of a bladder neck contracture are unlikely to respond well to collagen injection therapy [194].

Recently, collagen injections (Linerase^®^) have been proposed for the treatment of male genital *lichen sclerosus* and retrograde ejaculation. In the case of lichens sclerosus, it revealed to be safe and effective in 10 days and for up to 12 months [165]. Likewise, two injections of 6 mL of collagen (one per year) were revealed to be effective and complication free in cases of retrograde ejaculation [198]. 

Collagen-based injections were also found to be effective for the treatment of premature ovarian failure [212]. Indeed, umbilical cord mesenchymal stem cells loaded collagen formulation was found to be able to rescue overall ovarian function, evidenced by elevated estradiol concentrations, improved follicular development, and increased number of antral follicles [212]. Moreover, successful clinical pregnancy was achieved after the transplantation of the cell loaded collagen gel [212].

Thus, collagen injections seemed to be a simple, least morbid, cost-effective, and effective treatment for disease affecting the urinary apparatus, with low failure rates [123].

### 6.4. Gastrointestinal Apparatus

Injectable collagen has been shown to be effective in the management of gastrointestinal apparatus diseases such as glottic insufficiency [113,114,116,117,118,119,173,199,200,201,202,203], rectal fistula [153,154,156,157] and fecal incontinence [115,155,204]. 

Glottic dysfunctions due to glottic gap, atrophy, paresis, bowing, paralysis and scarring result in voice absence or alteration. The gold standard for the treatment of vocal fold disfunctions is represented by medialization laryngoplasty or arytenoid adduction, surgical treatments that could significantly improve glottal adduction and phonation. Recently, to reach a better postoperative voice in the long term, biomaterials injection (i.e., autologous fat, silicone, collagen, hyaluronic acid, carboxymethylcellulose) [116,224,225] has been additionally performed. However, autograft represent the known advantages of a double surgery, but means double surgery time and costs. Instead, xenografts are an attractive alternative for supplementing arytenoid adduction, because of their noninvasiveness, ready availability, and possibility to be performed under local anesthesia. Among them, collagen injectable formulations proved to be effective for vocal fold management. Patients treated with 1–2 mL of selected collagen injectable formulations (Koken^®^, AlloDerm^®^, Zyplast^®^) showed at least some improvement in vocal function after the treatment, according to the Grade, Roughness, Breathiness, Asthenia, Strain (GRBAS) scale, Maximum phonation time, Mean flow rate, Relative glottal area. In particular, perceptual and objective voice quality improvement (less weak and breathy) was registered, with an increase of the mean maximum phonation time from around 8–11 s to 13–15 s, and a reduction of the mean flow rate from 322–564 mL/s to 223–385 mL/s and of the glottal gap [113,114,200], for at least up to 2 years after operation [114]. Thus, from the moment in which the safety and efficacy of collagen injections for the treatment of the vocal cords was affirmed by Ford and Bless in 1993 [202], the injection of heterologous material started to be even more required, given the positive feedback and long-term results [118]. Although collagen injections were quite effective, and serious adverse events were rare [113,114,117,202], documented complications included local abscess, migration of the implant, hypersensitivity reactions, stiffening, fusiform collagen mass, nodules [116,173] principally related to the procedure and injection site [113]. Indeed, if properly injected, the complication rate after collagen injection would decrease [200]. 

Anal fistula is a tunnel that connects an infected cavity in the anus, to an opening on the skin. Usually, fistulas are surgically removed by fistulotomy, which is the gold standard procedure (37–98% success rate). However, complex fistula fistulotomy may result in variable degrees of anal sphincter apparatus impairment. Several alternative treatments were proposed and among them a trans anal rectal advancement flap represents the most effective treatment for complex anal fistulas allowing the successful closure of the internal opening. However, the recurrence rate is approximately 30% [157]. The interest in biomaterials use increased for their simple and repeatable application, preservation of sphincter integrity, and minimal patient’s discomfort [157]. Among biomaterials, fibrin glue and collagen injections were proposed. Fibrin glue was soon abandoned for its high rates of recurrences. Conversely, collagen injections (Permacol^®^) were revealed to be effective to treat anal fistula. In particular, no complications occurred and complete healing was reached after 3–15 months upon surgery [156,157,158]. The treatment success rate varies among studies, with a 56% of success rate at 12 months of follow up in a more recent study [156]. However, it should be mentioned that patients’ characteristics play a key role in the healing rate since a significant correlation with age was registered by Giordano et al. [156], with an increased chance of healing as age increased. While some authors confirmed the complete safety of the procedure and of the collagen injections [153,154,157,158], others registered middle-serious adverse events, including abscess (3%), bleeding (3%) and pain (7%) [156]. Although reports suggest that collagen injections are quite safe, minimally invasive, healing promoters for the sphincter-preserving procedure, and well tolerated by patients, further studies are needed for confirming their effectiveness in the treatment for complex anal fistulas.

Similarly, collagen injections were revealed to be quite effective for the treatment of fecal incontinence [115,155]. As reported by Stojkovic et al., after 2 months 5% of patients were completely continent, 58% had an improved incontinence score and 37% had no change or a worse score [115]. Healing was discovered to be strictly dependent on the incontinence etiology: a significant improvement of the incontinence score was indeed registered in case of idiopathic fecal incontinence and in older people while no improvements were observed in case of neuropathic or traumatic incontinence [115]. Despite the partial positive 1–2 years positive follow up, the disadvantage of collagen as filler agent is that degradation occurs over a period of 12–30 months [115] that obliges at least one repeat injection.

### 6.5. Others

New experimentations using collagen-based formulations were performed for non-standard clinical applications such as facial nerve rehabilitation after palsy [160,211], organ protection during thermal ablation [129], COVID-19 related hyperinflammation [161,162] (NCT04517162), artery aneurysms closure [128,213], blood volume augmentation [127,214] and the treatment of chronic ischemic heart diseases [226].

Given the absence of experiences with collagen-based injections in the field of facial palsy rehabilitation, the aim of a recent pilot randomized study was to test the short-term effectiveness of a collagen-based treatment (MD Neural^®^, MD Matrix^®^ and MD Muscle^®^) on patients complaining of long-standing facial nerve axonotmesis with the possible expectation of collagen redirecting and guiding reinnervation/reorganization processes [160,211]. Although the recovery outcomes are difficult to interpret because of the presence of several confounding factors (i.e., palsy etiology, time from disease onset, patients’ age, association of medical treatment), a significant improvement of both electrophysiological and questionnaire scores in the duration of voluntary activity was found in patients treated with in situ collagen injections [160,211].

Another recent application field for collagen-based injectable formulations is in the surrounding organ protection during tumor thermal ablation [129]. Organ protection is usually performed by using fluids (e.g., dextrose) or gas (e.g., CO_2_) displacement but because of their physical properties they distribute freely in the injection site and decrease the durability of separation. The injection of a highly viscous fibrillar collagen (Helitene^®^) focally interposed between the liver and adjacent structures prior to hepatic microwave ablation made the organ separation durable, low cost, well tolerated, facilitated hemostasis and healing besides making thermal ablation technically successful without complication [129].

A collagen-based injectable formulation was found to be a potential drug in the treatment of symptomatic COVID-19 patients for its immunomodulatory properties, in relation to IL-1β, IL-8, TNF-α, TNF-β1, IL-17, cyclooxygenase 1 (Cox-1), endothelial leucocyte adhesion molecule 1 (ELAM-1), vascular cell adhesion molecule 1 (VCAM-1), intercellular adhesion molecule 1 (ICAM-1) downregulation, tissues fibrosis reduction, and IL-10 and T cells upregulation. Intramuscular injection of collagen (Fibroquel^®^) was able to significantly decrease the interferon gamma-induced protein 10 (IP-10), IL-8, macrophage colony-stimulating factor (M-CSF), high-sensitivity C-reactive protein (hsCRP), D-dimer and lactate dehydrogenase (LDH) levels, in the first week of treatment [162] (NCT04517162). Moreover, collagen injections were associated with better oxygen saturation values and shortened symptom duration, extubation and reduced inflammation when compared to placebo [161,162] (NCT04517162). Thus, collagen-based injections were considered safe and well-tolerated and did not induce liver damage, infections, impairment of hematopoiesis or blood alterations [161,162] (NCT04517162).

Interestingly, collagen intravenous injections (Gelaspan^®^, Gelofusione^®^) were successful for blood volume expansion in cases of dehydration, illness, trauma or severe sepsis/septic shock related surgery and were found to be more effective in achieving hemodynamic stability in critically ill patients compared to standard plasma volume replacement products, with no side effects [127,214].

In cases of complications due to percutaneous transfemoral catheter procedures, vascular surgery is necessary after the failure of the ultrasound-guided compression repair attempt [128]. A less invasive method to percutaneously close a femoral artery pseudoaneurysm was found by injecting collagen and inducing clotting within the aneurysm, with a 98% success rate [128]. The hemostatic power of collagen is due to the fact that the collagen hydrogel forms an 3D network which triggers the hemostatic cascade (i.e., platelet aggregation, adherence, and activation) [213]. Moreover, upon contact with blood, the collagen expands its physical mass resulting in mechanical occlusion of the vessel puncture site and tissue tract [213].

Despite all the functional improvements that collagen is able to support in several diseases, neither improvement nor adverse events were observed in patients with chronic ischemic heart disease treated with mesenchymal stromal cells in a collagen gel vehicle compared with control patients and patients treated with mesenchymal stromal cells alone [226].

Thus, as emerged in this section, collagen-based injectable formulations can be very useful in the treatment of unresolved issues and open the way for new solutions and less invasive approaches. Based on this evidence, even more research has been performed and accordingly, even more clinical studies have been planned. Hence, besides the discussed clinical outcomes, several clinical studies aiming at improving functional recovery of liver in cases of decompensate cirrhosis (NCT02786017), brain in cases of intracranial hematoma (NCT02767817), erectile function in men with type I or II diabetes mellitus (NCT02745808), blood volume during surgery (NCT02808325, NCT01515397) and fluid retention in cases of breast cancer (NCT04637308) are ongoing (Table 4).

## 7. Adverse Reactions to Collagen-Based Injectable Implants

All types of fillers may trigger an early tissue response to the injected material. Regardless of the filler material, frequently reported side effects are bruising, redness, swelling, induration, erythema pain, tenderness, itching and, in the most severe cases, violaceous plaque and granulomas [227,228,229]. These side effects are usually mild and transient and resolve spontaneously after a short time. Only a few cases of severe and permanent complications have been registered. 

Although compared with other injectables collagen-based formulations have many advantages, it does not mean that they are absolutely safe. Indeed, severe and non-severe adverse reactions to collagen treatments may occur. To the best of our knowledge, based on harvested and available data on adverse reactions registered after collagen-based commercial product applications (Table 5), severe adverse events accounted for 8.2% (211 cases on 2587 patients), while mild adverse events accounted for about 5.3% (137 cases on 2587 patients) of those receiving the treatment. 

With a focus on collagen extraction sources, it emerged that severe adverse events accounted for 12.1% (211 cases on 1742 patients) and mild events for 3.8% (67 on 1742 patients) when bovine collagen was used. In particular, severe adverse events were addressed to the use of one collagen-based product that was Augment^®^, an injectable formulation composed of bovine collagen, β-tricalcium phosphate and recombinant human platelet-derived growth factor-BB [102] (NCT01305356, NCT00583375). Leaving aside the Augment^®^ severe adverse reactions (211 on 1742 procedures), the other analyzed bovine collagen-based products (i.e., ChondroGrid, Atelocell, Zyderm, Zyplast, Contigen, Gelofusine, Flowable wound matrix and Helitene) were not associated with such issues [88,113,114,116,118,127,129,142,187,188,190,214,230] (NCT02808325, NCT04637308, NCT02715466, NCT01515397, NCT02631356, NCT00868062). Since bovine collagen appeared to be safe, these events could be ascribable to other Augment components, without certainty. As regards mild adverse reactions, they were registered only when using Augment, Chondrogrid or Zyderm [101,106,118,142]. 

Porcine derived collagen-based products (i.e., Cartifil, Cartizol, Fibroquel, Permacol and MD products) revealed to not trigger severe adverse events (no cases on 751 procedures) and to be responsible for the 9.2% of mild adverse events (69 cases on 751 procedures) [131,132,133,134,135,136,138,140,141,143,145,146,153,154,155,156,157,160,161,162,182,204] (NCT02539030, NCT02539095, NCT04019782, NCT03323567, NCT02539082, NCT01528995, NCT04517162, NCT04353908). Mild adverse events could be due both to collagen type or to other components (i.e., glucose, CaCl, amino acids, vitamin B, fibrin glue for Cartifil/Cartizol; polyvinylpyrrolidone for Fibroquel) or to the injection procedure. However, data were not enough to identify the causes. Definitely though, the low mild adverse events rate of the MD product could be clearly ascribable to the presence of other bioactive compounds (such as calcium phosphate, rhododendron, arnica, hamemelis, silicon, iris, viola, cimifuga, citric acid, nicotinamide, hypericum, drosera, citrullus, ascorbic acid, magnesium gluconate, pyridoxine chlorhydrate, riboflavin, thiamine chlorhydrate) that had a strong impact on patients’ post intervention events. As regards Permacol, since it is not characterized by the presence of other components, adverse events triggered by its use could be attributed to collagen type, to the injection procedure, to the disease or to the patient specific response. In this case, available data do not allow clearly attribution of responsibility. However, mild adverse event usually resolved spontaneously or required minimal, not invasive intervention [131,140,141,156,160,162,204] (NCT04353908, NCT04517162, NCT01528995, NCT02539030). 

The third most used collagen type Is equine derived collagen, whose use is very recent and thus limited compared to bovine and porcine derived injectable products. Indeed, it has been reported to be used (i.e., Linerase, Savecoll-E) only on 94 patients, with no adverse events and only one registered mild reaction (1.1%) [60,164,165,166]. Thus, although this percentage seems to be very low compared to other products, the limited number of executed procedures with equine collagen prevented the assessment of this collagen type as safer. This consideration could be applied also for human collagen derived products (i.e., Cymetra, Dermologen) for which two severe and zero mild adverse events were registered on the only patients [173,202]. However, these data and these considerations are only indicative because not all studies reported participant number and adverse event occurrence.

As regards aesthetic applications, collagen injectables are generally considered as safe because serious adverse events that were not injection site related usually not occur [67]. Indeed, severe adverse events rate accounted for 0.1% of the total (2 cases on 2063 patients). In particular, severe adverse events occurred only when using porcine derived collagen Dermicol-P35 (with ribose as crosslinker) and RPC Pure Collagen (with ethylenediamine tetraacetic acid) [67,147]. However, two cases occurring on 780 injections were not enough to relate the adverse events to collagen type or other components. Contrarily, non-severe adverse reactions always occur (Table 3) and accounted for 28% (577 cases on 2063 patients). They may be categorized into early and late reaction [16,231]. Usually, injection site reactions were mild to moderate in severity and resolved in 1–2 weeks without sequelae [2,86], except for some rare cases. About 80% of participants had at least 1 injection site reaction after the initial injection [67]. This kind of adverse reaction is localized and may be associated with transient systemic symptoms on rare occasions [232]. Early complications occur immediately up to several days after treatment and completely auto-resolve in a few months, without treatment [217,227,229]. They can be divided into non-hypersensitive and hypersensitive reactions. Non-hypersensitive reactions, which can occur with any filler, include local injection site reactions (i.e., erythema, edema, pain, tenderness, bruising, itching), discoloration (i.e., redness, whiteness, or hyperpigmentation), infections (i.e., herpes virus reactivation or bacterial contamination), skin necrosis (vascular occlusion), and misplacement [231]. Hypersensitive reactions are due to the material and depend on patient immune system reactivity and hypersensitivity. Late complications occur after 2–12 months and consist in foreign body granulomatous reaction, granulomas, and abscess formation [231]. Among non-severe adverse events, pain (13.9%), nodule (11.0%), bruises (11.1%), edema (11.3%), erythema (17.8%), itching (5.8%), swelling (6.0%), tenderness (3.0%), lumpiness (1.4%), induration (12.0%), discoloration (5.3%) were the most common. Very rare were cold sores, infections, blistering, papules, and hemorrhages (>0.5%). Contrary to what might be expected, allergic reactions occurred only in 0.1% of cases.

In terms of collagen extraction source, bovine (48%) and porcine (38%) derived formulations were the most used, followed by human (11%) and equine derived (4%). Accordingly, Dermicol-P35 and Zyplast were the most used products, followed by Therafil, Artecol, Koken, Isolagen therapy, CosmoPlast, Nithya, RPC Pure-Collagen, Permacol, Sunmax FacialGain, and CosmoDerm. Non severe events were registered to happen with all collagen types, except for equine derived collagen-based formulations. Indeed, bovine and swine derived collagen-based formulations triggered 12.5% and 11.8% non-severe events (257 and 244 cases on 2063 injections, respectively), followed by human derived with about 3.7% (76 cases on 2063 injections). Equine collagen injectables were revealed to be adverse events free but it should be taken into account that reported data were referred to only one study performed on 72 people [163]. Thus, Nithya, RPC Pure Collagen and Artecol reported no adverse events [67,77,163]. The non-severe adverse events rate was reported to be of 39.2% for Dermicol-P35 (213 cases on 544 injections) [2,147,148,149,207,209,210] (NCT00891774, NCT00929071, NCT00911872), 94.7% for Permacol (18 cases on 19 injections) [95], while for the others the rate was about 7–36%. In particular, it was 32.8% for Zyplast (13/187 injections) [96] (NCT00876265), 20.2% for Koken (23/114 injections) [87], 29.1% for the Isolagen therapy (32/110 injections) (NCT00655356), 36.4% for Cosmoplast (43/118 injections) [205,206] (NCT00444210, NCT00444353), 33.3% for Cosmoderm (1/3 injections) [217] (NCT01212809) and 7% for Therafil, (13/187 injections) [87] (NCT01060943). In this case, the relatively low number of executed procedures prevented the assessment of product safety profiles and their comparison.

Although non-severe adverse reactions are neither life nor health threatening and thus are not of medical significance, they are cosmetically unacceptable. Nowadays, several tricks and improvements of the injection techniques have been made in order to avoid reactions caused by materials and procedures as much as possible [233]. Hypersensitive reactions are historically defined as the most common. Although it is rare (3% of the world population), some individuals develop allergic reactions to injected products when the body responds with an exaggerated immune response to a foreign substance. Allergic reactions generally occur within minutes of exposure, but delayed hypersensitivity can occur several months or years after injection [9]. Allergy to bovine derived collagen is genetically regulated by the lack of the HLA-DR4 antigen [234]. To avoid allergic reaction, skin testing now is mandatory. However, despite skin testing, hypersensitivity can occur in 1–6% of single skin test negative patients and in about 0.5% of double skin test negative patients [16,227,228,229]. Thus, a double skin testing is suggested before soft tissue augmentation [228,235]. Although double skin testing does not eliminate all adverse events, most of them were avoided because the great majority of adverse reactions occurred on the first injection session after a single skin test [232]. Collagen antigenicity is related to its molecular structure and is linked to its antigenic determinants that are located on the triple helix (i.e., dependent on the helix conformation), the polypeptide sequence (i.e., independent of the helices organization) and terminal (i.e., telopeptides) dependent [71,236,237,238]. However, it must be underlined that while collagen antigenicity has been attributed mostly to the terminal telopeptides, the location of the major antigenic sites depends on the specific donor/recipient species pair [71]. Alternatively, human collagen-based fillers offer a solution for their theoretical zero risk of allergic reaction. Nevertheless, erythema and hypersensitivity to human collagen was registered [217].

Foreign body granulomatous reaction and granulomas occurred in 0.01% of cases after 6–9 months after the treatment [231]. However, it must be taken into account that most of this kind of complication occurred with Artecoll^®^/Artefill^®^, probably due to the reaction to the poly(methyl methacrylate) microspheres rather than the collagenous component. However, the late adverse reactions to poly(methyl methacrylate) microspheres together with bovine collagen may have increased the immune system response. 

Apart from the selected collagen formulation, as with any surgical or minimally invasive procedure, the result obtained with the injection therapy heavily depends on proper patient selection, expertise in performing the procedure, adequate knowledge of facial or other site’s anatomy, and use of specialized equipment [13,123]. Only recently, the development of adequate implantation protocols permitted re-evaluation of collagen-based injectable therapies as a minimally invasive and effective strategies for the treatment of different types of diseases. Indeed, as preparation and administration techniques have become increasingly standardized, the frequency of post-injection complications has also decreased. Moreover, the selection of the appropriate filler, which depends on patient factors, including degree of volume loss, disease, age, cost, preference, and surgical candidacy was revealed to be crucial for the implant success [1], underscoring the need for product-specific training. Regardless of materials safety, appropriate handling and adequate experience are mandatory for minimizing the risk of complications and achieving the desired effect. An accurate guide on how to avoid and treat dermal filler complications has been developed by Lemperle et al. [233].

Swelling and bruising, which usually resolves within 4–10 days [163], could be attenuated by icing the area prior to treatment or by avoiding aspirin-containing compounds and anticoagulants, nonsteroidal anti-inflammatory drugs, and various vitamin supplements (e.g., vitamin E, fish oils) for 7–10 days before the procedure [152,231]. Only one severe bruising was reported after 1-week follow-up and resolved after 4 weeks [67]. A case of mild induration after 4 weeks resolved in 6–12 weeks [67].

The gauge of the needle, that depends on both the viscosity of the filler and the size of the particle, directly greatly contributes to the extent of superficial trauma and infections. Larger needle size can lead to a larger epithelial tear and greater disruption of dermal structures, with subsequent capillary leakage, edema, inflammation and sometimes infections [9].

Infection and abscess formation are rare complication of collagen fillers and can occur early on or can be delayed for several weeks to months after injection [231]. Early infections could be prevented by cleaning the treatment area with an antiseptic agent (e.g., isopropyl alcohol, chlorhexidine) while late infections could be treated by broad-spectrum antibiotics or anti-viral prophylaxis [231]. Herpes was registered in 1 case on approximately 15,000 injections [168]. Abscess formation is also rare (4 out of 10,000 patients) and occurs between 7 days to 22 months after treatment and may persist for weeks and periodically recur for months [16].

The occurrence of complications is also dependent on the injection site. Sensitive areas, such as around the mouth or beneath a muscle, heighten the risks for unwanted side effects. A bluish discoloration is associated with vascular injury due to injection. Vascular interruption also heightens the risk for local necrosis. Skin necrosis from mechanical disruption or occlusion of the vascular supply can rarely occur (9 out of 100,000 patients) [16]. Iatrogenic blindness is a rare but possible risk caused by misplaced intravascular injection. The risk is correlated to the complex vascular anatomy of the face interconnecting the extracranial and intracranial vascular network [13]. This tragic complication occurs when the filler is wrongly injected in the ophthalmic artery. Nowadays, several precautions can be taken to avoid necrosis. When injecting, attention should be paid to avoiding arteries, to aspirate before injecting, to use low volumes of products over more sessions as opposed to using high volumes over one session and to use only products that are manufactured for more superficial placement [231]. Moreover, warm compresses, massage, and tapping on the area were revealed to facilitate vasodilation and blood flow [231]. 

Improper distribution of injected products can also lead to lumps and nodules post-injection, besides facial shape deformity and asymmetry [9]. Denton et al. reported that it is very important to massage the product immediately after placement to mold and smooth the contour [152]. In case of over-injected or under-injected areas, palpation and massage should be performed to evenly distribute the material [86]. 

**Table 5:** Collagen injection specifications (number, volume and time), adverse events recurrence (severe and non-severe) and other details from most recent clinical trials on musculoskeletal, gastro-intestinal, urinary, circulatory apparatus and others from 2000 to 2022.

Application	Product	Disease	Injection Specification			Observation Time (Weeks)	Participants (n)	Adverse Events		Ref.
			Number (n)	Volume (mL)	Inj./Time (w)			Severe	Mild	
Musculoskeletal apparatus	Augment	Non fused foot and ankle	1	3–6	1	36	14	0	36	[108]
			1	6–9	1	12	7	0	0	[110]
			1	1–9	1	52	26	0	0	[109] NCT00583375
			1	n. d.	1	52	132	75	27	[101], NCT01305356
		Arthritis	1	1–9	1	52	394	136	n. d.	[102], NCT00583375
	Cartifill	Knee cartilage	1	1	1	96	52	0	5	[139], NCT02539030
		Cartilage lesion	1	3	1	6	1	0	0	[138]
	CartiZol	Osteoarthritis	1	3	1	24	101	0	7	[140]
		Chondromalacia, osteoarthritis	1	n. d.	1	n. d.	n. d.	n. d.	n. d.	NCT02539095
	ChondroGrid	Osteoarthritis	3	2	0.5/w (2 w), 0.25/w (1 w)	24	70	0	3	[142]
			3	6		32	20	0	0	[112]
	Fibroquel	Osteoarthritis	3	1.5	1/w	24	n. d.	n. d.	n. d.	NCT04019782
			5	2	1/w	24	10	0	n. d.	[182]
	Linerase	Gingival recession	3	14	1.5/w	n. d.	18	0	0	[167]
	MD-Hip	Osteoarthritis	1	2	1	96	24	0	1	[131]
	MD-Knee, MD-Muscle	Osteoarthritis	10	n. d.	2/w (2 w), 1/w (6 we)	12	30	0	0	[133]
	MD-Lumbar, MD-Muscle, MD-Neural	Lumbar spine pain	5	20	2/w (2 w), 1/w (1 w)	6	73	0	0	[134]
	MD-Knee, MD-Matrix	Sprained knee	6	n. d.	2/w	3	10	0	0	[143]
	MD-Muscle or MD-Matrix	Piriformis syndrome	1–3	n. d.	n. d.	n. d.	28	0	0	[136]
	MD-Lumbar, MD-Ischial	Chronic pain due to arthrosis, myalgia	1	1	1	10	71	0	0	[132]
	MD-Lumbar, MD-Matrix	Back pain	10	n. d.	2/w (2 w), 1/w (6 w)	8	1	0	0	[135]
	MD-Lumbar, MD-Muscle, MD-Matrix	Lumbar joint block	9	n. d.	2/w (2 w), 1/w (5 w)	7	1	0	0	[135]
	MD-Muscle, MD-Neural	Muscle pain	1	1	1	10	53	0	0	[132]
	MD Shoulder	Calcific supraspinatus tendinitis	4	n. d.	1/w	6	10	0	0	[145]
	MD-Shoulder, MD-Muscle	Shoulders periarthritis	10	n. d.	2/w (2 w), 1/w (6 w)	8	22	0	0	[146]
	MD Muscle	Myofascial pain	2	2	1/w	2	18	0	9	[130], NCT03323567
	RegenSeal	Plantar fasciitis	n. d.	n. d.	n. d.	n	n. d.	n. d.	n. d.	NCT02539082
		Rotator cuff tears	1	1	1	48	62	0	0	[141]
	n. d.	Rotator cuff tears	4	8	1/w	72	1	0	0	[183]
	n. d.	Osteoarthritis	1	2	1	24	n. d.	n. d.	n. d.	NCT04998188
Gastro-intestinal apparatus	Atelocell	Vocal folds paralysis	1	0.5–1.3	1	12	155	0	0	[114]
			1	0.5–1.3	1	12	40	0	0	[113]
	Cymetra	Vocal folds paralysis	1	1	1	4	8	0	0	[203]
			1	n. d.	1	2	1	1	0	[173]
	Dermologen	Laryngoplasy	2	n. d.	0.7/w	4	1	1	0	[173]
	Permacol	Fecal incontinence	1	1.5	1	6	28	0	8	[204], NCT01528995
			n. d.	n. d.	n. d.	48	14	0	0	[155]
		Anal fistula	1	n. d.	1	36	11	0	0	[157]
			1	n. d.	1	48	28	0	7	[156]
			1	n. d.	1	12	1	0	0	[153]
		Rectovaginal fistula	1	n. d.	1	8	1	0	0	[154]
	Salvecoll-E	Anorectal fistula	1	2	1	48	70	0	0	[60]
	Zyderm	Laryngeal paralysis	1	0.2–0.5	1	24	7	0	1	[118]
	Zyplast	Laryngoplasy	1	n. d.	1	24	100	0	0	[116]
urinary system	Contigen	Sphincter incontinence	1–5	2–4	0.05/w	84	63	0	0	[188]
		Urethra hypermobility	1–4	14	1	172	58	0	0	[187]
		Neurogenic bladder dysfunction	1–4	n. d.	n. d.	192	20	0	0	[190]
	Linerase	Lichen sclerosus	6	27	2/week (2 w), 1/8 w	8	1	0	1	[165]
	n. d.	Retrograde ejaculation	2	6	1/year	96	1	0	0	[198]
	n. d.	Bilateral vesicoureteral reflux	1	2.5	1	144	1	1	0	[223]
	n. d.	Stress urinary incontinence	1–2	n. d.	0.25/w	36	40	0	1	[185]
	n. d.	Premature ovarian failure	1	n. d.	1	12	8	0	0	[212], NCT02644447
	n. d.	Erectile disfunction	n. d.	n. d.	n. d.	n. d.	n. d.	n. d	n. d.	NCT02745808
Circulatory system	Gelofusine	Blood volume	1	n. d.	1	n. d.	n. d.	n. d.	n. d.	NCT02808325
		Fluid retention	1	500	1	n. d.	n. d.	n. d.	n. d.	NCT04637308
		Severe sepsis	1	500	1	13	608	n. d.	n. d.	[127], NCT02715466
		Abdominal surgery	n. d.	n. d.	n. d.	n. d.	n. d.	n. d.	n. d.	NCT01515397
		Blood volume	1	10 mg/k	1	n. d.	5	0	0	[230], NCT02631356
		Blood volume	1	1 L	1	4 h	12	0	0	[214], NCT00868062
others	Fibroquel	COVID-19 due hyperinflammatory syndrome	10	15	14/w (3 days), 12/w (4 days)	12	45	0	33	[162], NCT04517162
			7	10.5	7/w	1	35	0	0	[161], NCT04517162
	Flowable Wound Matrix	Hand scar due to severe burns	1	3–6	1	24	8	0	0	[88]
	Helitene	Organ protection during ablation	1	10–30	1	12	3	0	0	[128]
	Linerase	Vitiligo	6	27	0.5/w	12	5	0	0	[164]
	MD Neural, MD Matrix, MD Muscle mix	Facial nerve palsy	16	13	2/w	8	21	0	8	[160], NCT04353908
	n. d.	Decompensated cirrhosis	n. d.	n. d.	n. d.	n	n. d.	n. d.	n. d.	NCT02786017
	n. d.	Brain injury	1	n. d.	1	n. d.	n. d.	n. d.	n. d.	NCT02767817
	n. d.	Ischemic cardiomyopathy	1	n. d.	1	48	50	1, heart failure	0	[226], NCT02635464

## 8. Regulation

Resorbable injectable soft tissue fillers can be classified as medical devices, medicinal products, or cosmetic products. Injectable formulations are defined as medical devices if their therapeutic effect comes from their intrinsic structure, because their physical, chemical, or mechanical effects are the primary mechanism of action for their therapeutic function. The addition of any cells or cell-stimulating therapeutics into the injectable medical device results in their classification as medicinal products and in the following of other regulations. Indeed, medicinal product regulations require a more thorough investigation of the biocompatibility and therapeutic effect before approval for clinical application. Although medicinal products would be more effective, the translational barriers and the time before patients can benefit from them strongly increase.

In the United States, resorbable injectable soft tissue fillers have long been classified as medical devices while in Europe, dermal fillers have been marketed as medical devices, medicinal products, or cosmetics until now. However, with the entry into force of the new Medical Device Regulation (MDR) 2017/745 on 26 May 2021, all fillers (both for cosmetic and for medical purposes) are classified as class III risk medical devices. This means that all injectable products must be CE certified by a notified body if marketed after 26 May 2020. Thus, manufacturers required documentation including a device master record (technical documentation) and product clinical evaluation in accordance with MEDDEV 2.7/1 as well as an appropriate quality management system according to the Medical Devices Directive (MDD) ISO 13485. As regards injectable soft tissue fillers for cosmetic purposes, because of the absence of an intended medical purpose, they do not require a clinical efficacy investigation, but they are subject to a clinical evaluation regarding safety. Additionally, for materials of animal origin, such as collagen, manufacturers have to comply also with Regulation EU 722/2012.

## 9. Concluding Remarks

Forerunner fillers were plagued by frequent unwanted side effects and serious complications (i.e., migration of injected filler, granulomatous inflammation, tissue necrosis, and hypersensitivity reactions). With the advancement of research, a new generation of fillers has been developed that have overcome some of the many existing earlier problems. The steps forward regarding safety and the refinement of injection techniques brought an exponential increase in and use of soft tissue filler products and procedures. This growth was fueled by the increased availability of new dermal filler products and by their improved safety profiles.

Injectable systems hold great promise in tissue engineering applications as they can potentially provide for an adequate temporal environment for the injured site regeneration, as well as delivering water soluble drugs, growth factors and cells for better outcomes. Thus, the injectable formulations must have both structural (i.e., filling role) and biological (i.e., pro-regenerative action) impermanent functions. In particular, the hydrogel should not only structurally support tissue regeneration but also stimulate its regeneration and be gradually digested and replaced by the newly synthetized tissue, resulting in a new functional tissue. The success of any injectable system is strongly determined by the framework the hydrogel provides. The 3D network should provide mechanical support compliant with the injured tissue, adequate pore size and interconnectivity to allow mass transport and regenerative processes, and eventually, must provide for the controlled release of bioactive molecules. In clinical setting, injectable materials hold the promise of being an effective minimally invasive treatment for mild-severe defects. The delivery of cells, bioactive factors, and support materials via an injectable system within the context of an endoscopic, arthroscopic, laparoscopic, or radiologically guided procedure is feasible and potentially successful. With the growing knowledge and technology in biomedical and materials sciences, the innovation of injectable biomaterials to fulfill unmet clinical needs is expected to thrive in the near future [14]. Indeed, a high number of bioactive injectable biomaterials have been developed and approved for clinical use.

Among them, multifunctional collagen products are effective in some clinical applications. However, there are some points to be clarified and obstacles to overcome in order to develop disease specific products. As the outcomes of research move toward clinical translation, the elucidation of the mechanisms of interactions between an injectable biomaterial and its surroundings is necessary to reach optimal material performance. However, the interaction between the host tissue and the material is unknown due to the lack of accurate and adequate in vivo evaluation. The scarcity of tools for the in vivo evaluation of injectable biomaterials has posed numerous difficulties in fully understanding injection consequences [14] but, nowadays, new advanced investigation techniques such as cone beam and micro computed X-ray tomography, immunohistochemistry, small-angle X-ray scattering, X-ray diffraction, and the more recent fluorescent labelling of abundant reactive entities, optical photothermal infrared microscopy and infrared atomic force microscopy, fluorescence lifetime imaging microscopy and Raman spectroscopy will allow us to overcome this issue and deeply understand the material’s action mechanism over time [3]. These techniques will also allow us to tune the properties of injectable materials according to patient specific disease requirements and comorbidities in order to develop personalized therapies. Moreover, the deep in vivo investigation of the material-tissue interaction will allow us to overcome another important issue of injectable formulations, that is, the effectiveness of mass transport. Clinically available collagen injectables efficacy is hindered by the absence of nutrients necessary to support cell regenerative processes that could be responsible for delayed and deficient integration with the host tissue, especially in the case of large defect regions [68]. To overcome these limits, Alnojeidi et al. developed a new in situ cross-linkable injectable formulation of cross-linked bovine type I collagen, chondroitin sulfate and polyvinyl alcohol, that contains the optimum concertation of necessary amino acids, vitamins, and minerals required for cell growth and proliferation [68].

In addition, the high costs associated with the development and manufacture of medical-grade injectable biomaterials (i.e., basic and applied research on medical device design, raw material extraction, material properties assessment, sterile device production, package and storage condition assessment) or with the incorporation of therapeutic agents are another hindrance to be overcome [14]. Sensitivity analyses showed that surgery would be less costly and more successful than collagen injection if the postoperative length of hospital stay was reduced to 1 day or if the number of injections required to treat patients were more than two for treatment successes and more than four for treatment failures [186]. Endoscopic injection of collagen is effective in many cases, but its cost effectiveness depends on the number of re-injections required. In the treatment for vesicoureteral reflux single collagen injections were very effective and may effectively reduce health care management costs of about $7544 per renal unit (collagen injection cost: $1599, reimplantation cost: $9144) [239]. In the treatment of the stress urinary incontinence, collagen injection is more cost effective than surgery if one application resolves the problem [123]. Instead, surgery (i.e., artificial genitourinary sphincter placement) is more cost effective than collagen injection when more than three collagen injections are required (collagen injection cost: $4300–6021; artificial genitourinary sphincter placement $11,933–15,400) [123,240]. In the case of aesthetic surgery, fillers would be less costly than surgical rhytidectomy ($15,181) in cases of small facial area. In cases of large volume, the medical cost for surgery would be the lowest cost option among the other treatments over the course of several years [1].

Indeed, when developing a new injectable, materials factors such as product cost, scalability and maneuverability should be considered together with safety and quality profiles before proceeding with its pre-clinical and clinical evaluation. Many promising collagen-based materials have been designed and intensely investigated from the physical, chemical, mechanical, morphological and biological (both in vitro and in vivo) point of view but did not attain clinical translation. The consideration of the clinical potential of the material is nowadays mandatory to receive the regulation body approval besides expecting its clinical success [3]. This approach will reduce the tremendous discrepancy between the huge quantity of academic research and the number of products that have been clinically translated [3].

Therefore, much research still needs to be carried out before minimally invasive strategies equal or surpass in terms of effectiveness the currently performed surgical procedures. However, the complete replacement of time-consuming and costly surgeries with injections does not seem to be so far away. In fact, even more collagen-based products are demonstrating their effectiveness in one or more sessions and in various injured body structures. Furthermore, actual preclinical and clinical research is not only confirming their assessed efficacy, but it is improving both formulations and injection techniques, as well as testing them for new, challenging, unresolved diseases.

In achieving this ultimate goal, the collaboration and transparency between researchers, clinicians, patients and companies has proved to be the only constructive way to successfully develop innovative and functional products capable of truly improving human health and making such treatments viable on a large-scale, accessible to the majority of the population and offering patients a long-term quality of life.

## Figures and Tables

**Table 1:** Most important world companies producing clinical grade collagen and related extraction sources.

Animal Source	Extraction Tissue	Company
Equine	Tendon	Euroresearch S.r.l. (Milan, Italy) www.euroresearch.it, accessed on 14 February 2023
	Tendon	Opocrin Spa (Formigine, Italy) www.opocrin.it, accessed on 14 February 2023
	Tendon	Typeone Biomaterials S.r.l. (Calimera, Italy) www.typeone.it, accessed on 14 February 2023
Bovine	Corium, tendon, membranes	Bovine collagen products (Branchburg, NJ, USA) www.bovinecollagenproducts.com, accessed on 14 February 2023
	Corium, tendon	Collagen solution (Eden Prairie, MN, USA) www.collagensolutions.com, accessed on 14 February 2023
	n. d.	Royal DSM (Heerlen, The Netherlands) www.dsm.com, accessed on 14 February 2023
	Tendon	Integra LifeScience Corp. (Princeton, NJ, USA) www.integralife.com, accessed on 14 February 2023
	Dermis	Koken Co., Ltd. (Tokyo, Japan), www.kokenmpc.co.jp, accessed on 14 February 2023
	Dermis	Devro Plc (Moodiesburn, UK) www.devro.com, accessed on 14 February 2023
	Tendon	Getinge (Göteborg, Sweden) www.getinge.com, accessed on 14 February 2023
	Dermis	Symatese (Chaponost, France) www.symatese.com, accessed on 14 February 2023
	Hide	Advanced Biomatrix (Carlsbad, CA, USA) www.advancedbiomatrix.com, accessed on 14 February 2023
Swine	Skin	Ubiosis (Gyeonggi-do, Republic of Korea) www.ubiosis.com, accessed on 14 February 2023
	Skin	Botiss Biomaterials GmbH (Zossen, Germany) www.botiss-dental.com, accessed on 14 February 2023
Jellyfish	n. d.	Jellagen (Cardiff, UK) www.jellagen.co.uk, accessed on 14 February 2023
Plant	Leaves	CollPlant (Rehovot, Israel) www.collplant.com, accessed on 14 February 2023

**Table 4:** New applications of collagen based injectable formulations.

Formulation	Study Aim	Status	Outcomes	ClinicalTrials.gov Identifier
Injectable Collagen Scaffold^TM^ HUC-MSCs	Improvement of erectile function in men with diabetes	Unknown	n. d.	NCT02745808
Injectable Collagen Scaffold^TM^ HUC-MSCs	Improvement of liver function in cases of decompensated cirrhosis	Unknown	n. d.	NCT02786017
Injectable Collagen Scaffold^TM^ MCSs	Improvement of functional brain recovery in cases of brain injury	Unknown	n. d.	NCT02767817
Gelofusine	Fluid retention prevention in patients with breast cancer	Completed	n. d.	NCT04637308
Gelofusine	Improvement of blood volume in patients scheduled for abdominal or pelvic surgery	Completed	n. d.	NCT02808325
Gelofusine	Improvement of blood volume for intravascular volume compensation during surgery	Completed	n. d.	NCT01515397

## Data Availability

Not applicable.

## References

[1] Go B.C., Frost A.S., Friedman O. (2021). Using Injectable Fillers for Midface Rejuvenation. Plast. Aesthetic Res..

[2] Solish N.J. (2010). Assessment of Recovery Time for the Collagen Products Dermicol-P35 27G and 30G. J. Am. Acad. Dermatol..

[3] Øvrebø Ø., Perale G., Wojciechowski J.P., Echalier C., Jeffers J.R.T., Stevens M.M., Haugen H.J., Rossi F. (2022). Design and Clinical Application of Injectable Hydrogels for Musculoskeletal Therapy. Bioeng. Transl. Med..

[4] Kretlow J.D., Young S., Klouda L., Wong M., Mikos A.G. (2009). Injectable Biomaterials for Regenerating Complex Craniofacial Tissues. Adv. Mater..

[5] Béduer A., Genta M., Kunz N., Verheyen C., Martins M., Brefie-Guth J., Braschler T. (2022). Design of an Elastic Porous Injectable Biomaterial for Tissue Regeneration and Volume Retention. Acta Biomater..

[6] Eppley B.L., Dadvand B. (2006). Injectable Soft-Tissue Fillers: Clinical Overview. Plast. Reconstr. Surg..

[7] Buck D.W., Alam M., Kim J.Y.S. (2009). Injectable Fillers for Facial Rejuvenation: A Review. J. Plast. Reconstr. Aesthetic Surg..

[8] Requena L., Requena C., Christensen L., Zimmermann U.S., Kutzner H., Cerroni L. (2011). Adverse Reactions to Injectable Soft Tissue Fillers. J. Am. Acad. Dermatol..

[9] Luebberding S., Alexiades-Armenakas M. (2012). Safety of Dermal Fillers. J. Drugs Dermatol..

[10] Cheng L., Sun X., Tang M., Jin R., Cui W., Zhang Y.-G. (2016). An Update Review on Recent Skin Fillers. Plast. Aesthetic Res..

[11] Ginat D.T., Schatz C.J. (2013). Imaging Features of Midface Injectable Fillers and Associated Complications. Am. J. Neuroradiol..

[12] Lemperle G. (2007). Injectable Dermal Fillers—Resorbable or Permanent?. Aesthetic Surgery of the Facial Mosaic.

[13] Oranges C.M., Brucato D., Schaefer D.J., Kalbermatten D.F., Harder Y. (2021). Complications of Nonpermanent Facial Fillers: A Systematic Review. Plast. Reconstr. Surg. Glob. Open.

[14] Zhou H., Liang C., Wei Z., Bai Y., Bhaduri S.B., Webster T.J., Bian L., Yang L. (2019). Injectable Biomaterials for Translational Medicine. Mater. Today.

[15] Attenello N.H., Maas C.S. (2015). Injectable Fillers: Review of Material and Properties. Facial Plast. Surg..

[16] Cockerham K., Hsu V. (2009). Collagen-Based Dermal Fillers: Past, Present, Future. Facial Plast. Surg..

[17] Silvipriya K.S., Krishna Kumar K., Bhat A.R., Dinesh Kumar B., John A., Lakshmanan P. (2015). Collagen: Animal Sources and Biomedical Application. J. Appl. Pharm. Sci..

[18] Avila Rodriguez M.I., Rodriguez Barroso G.L., Sanchez M.L. (2018). Collagen: A Review on Its Sources and Potential Cosmetic Applications. J. Cosmet. Dermatol..

[19] Gallo N., Natali M.L., Sannino A., Salvatore L. (2020). An Overview of the Use of Equine Collagen as Emerging Material for Biomedical Applications. J. Funct. Biomater..

[20] Salvatore L., Gallo N., Natali M.L., Terzi A., Sannino A., Madaghiele M. (2021). Mimicking the Hierarchical Organization of Natural Collagen: Toward the Development of Ideal Scaffolding Material for Tissue Regeneration. Front. Bioeng. Biotechnol..

[21] Sandri M., Tampieri A., Salvatore L., Sannino A., Ghiron J.H.L., Condorelli G. (2010). Collagen Based Scaffold for Biomedical Applications. J. Biotechnol..

[22] Lee C.H., Singla A., Lee Y. (2001). Biomedical Applications of Collagen. Int. J. Pharm..

[23] Dong C., Lv Y. (2016). Application of Collagen Scaffold in Tissue Engineering: Recent Advances and New Perspectives. Polymers.

[24] Chattopadhyay S., Raines R.T. (2014). Review Collagen-Based Biomaterials for Wound Healing. Biopolymers.

[25] Sorushanova A., Delgado L.M., Wu Z., Shologu N., Kshirsagar A., Raghunath R., Mullen A.M., Bayon Y., Pandit A., Raghunath M. (2019). The Collagen Suprafamily: From Biosynthesis to Advanced Biomaterial Development. Adv. Mater..

[26] Gelse K., Pöschl E., Aigner T. (2003). Collagens—Structure, Function, and Biosynthesis. Adv. Drug Deliv. Rev..

[27] Ricard-Blum S. (2011). The Collagen Family. Cold Spring Harb. Perspect. Biol..

[28] Parvizi J., Kim G.K. (2010). Collagen. High Yield Orthopaedics.

[29] Goldberga I., Li R., Duer M.J. (2018). Collagen Structure–Function Relationships from Solid-State NMR Spectroscopy. Acc. Chem. Res..

[30] Owczarzy A., Kurasinski R., Kulig K., Rogoz W., Szkudlarek A., Maciazek-Juczyk M. (2020). Collagen—Stucture, Properties and Applications. Eng. Biomater..

[31] Arseni L., Lombardi A., Orioli D. (2018). From Structure to Phenotype: Impact of Collagen Alterations on Human Health. Int. J. Mol. Sci..

[32] Wang H. (2021). A Review of the Effects of Collagen Treatment in Clinical Studies. Polymers.

[33] Meyer M. (2019). Processing of Collagen Based Biomaterials and the Resulting Materials Properties. Biomed. Eng. Online.

[34] Shoulder M.D., Raines R.T. (2009). Collagen Structure and Stability. Annu. Rev. Biochem..

[35] Amirrah I.N., Lokanathan Y., Zulkiflee I., Wee M.F.M.R., Motta A., Fauzi M.B. (2022). A Comprehensive Review on Collagen Type I Development of Biomaterials for Tissue Engineering: From Biosynthesis to Bioscaffold. Biomedicines.

[36] Birk D.E., Brückner P. (2011). Collagens, Suprastructures, and Collagen Fibril Assembly. The Extracellular Matrix: An Overview.

[37] Salvatore L., Gallo N., Aiello D., Lunetti P., Barca A., Blasi L., Madaghiele M., Bettini S., Giancane G., Hasan M. (2020). An Insight on Type I Collagen from Horse Tendon for the Manufacture of Implantable Devices. Int. J. Biol. Macromol..

[38] Ignat’eva N.Y., Danilov N.A., Averkiev S.V., Obrezkova M.V., Lunin V.V., Sobol E.N. (2007). Determination of Hydroxyproline in Tissues and the Evaluation of the Collagen Content of the Tissues. J. Anal. Chem..

[39] Bou-Gharios G., Abraham D., de Crombrugghe B. (2020). Type I Collagen Structure, Synthesis, and Regulation. Principles of Bone Biology.

[40] Kadler K.E., Holmes D.F., Trotter J.A., Chapman J.A. (1996). Collagen Fibril Formation. Biochem. J..

[41] Collins C.J., Andriotis O.G., Nedelkovski V., Frank M., Katsamenis O.L., Thurner P.J. (2019). Bone Micro- and Nanomechanics. Encyclopedia of Biomedical Engineering.

[42] Terzi A., Gallo N., Bettini S., Sibillano T., Altamura D., Madaghiele M., de Caro L., Valli L., Salvatore L., Sannino A. (2020). Sub- and Supramolecular X-Ray Characterization of Engineered Tissues from Equine Tendon, Bovine Dermis and Fish Skin Type-I Collagen. Macromol. Biosci..

[43] Terzi A., Gallo N., Bettini S., Sibillano T., Altamura D., Campa L., Natali M.L., Salvatore L., Madaghiele M., de Caro L. (2019). Investigations of Processing–Induced Structural Changes in Horse Type-I Collagen at Sub and Supramolecular Levels. Front. Bioeng. Biotechnol..

[44] von der Mark K. (2006). Structure, Biosynthesis and Gene Regulation of Collagens in Cartilage and Bone. Dynamics of Bone and Cartilage Metabolism.

[45] Exposito J.-Y., Cluzel C., Garrone R., Lethias C. (2002). Evolution of Collagens. Anat. Rec..

[46] Chu M.-L., de Wet W., Bernard M., Ding J.-F., Morabito M., Myers J., Williams C., Ramirez F. (1984). Human Proα1(I) Collagen Gene Structure Reveals Evolutionary Conservation of a Pattern of Introns and Exons. Nature.

[47] Fidler A.L., Boudko S.P., Rokas A., Hudson B.G. (2018). The Triple Helix of Collagens—An Ancient Protein Structure That Enabled Animal Multicellularity and Tissue Evolution. J. Cell Sci..

[48] Exposito J.-Y., Valcourt U., Cluzel C., Lethias C. (2010). The Fibrillar Collagen Family. Int. J. Mol. Sci..

[49] Madaghiele M., Salvatore L., Sannino A. (2014). Tailoring the Pore Structure of Foam Scaffolds for Nerve Regeneration.

[50] Salvatore L., Madaghiele M., Parisi C., Gatti F., Sannino A. (2014). Crosslinking of Micropatterned Collagen-Based Nerve Guides to Modulate the Expected Half-Life. J. Biomed. Mater. Res. A.

[51] Madaghiele M., Calò E., Salvatore L., Bonfrate V., Pedone D., Frigione M., Sannino A. (2016). Assessment of Collagen Crosslinking and Denaturation for the Design of Regenerative Scaffolds. J. Biomed. Mater. Res. A.

[52] Salvatore L., Calò E., Bonfrate V., Pedone D., Gallo N., Natali M.L., Sannino A., Madaghiele M. (2021). Exploring the Effects of the Crosslink Density on the Physicochemical Properties of Collagen-Based Scaffolds. Polym. Test..

[53] Parisi C., Salvatore L., Veschini L., Serra M.P., Hobbs C., Madaghiele M., Sannino A., di Silvio L. (2020). Biomimetic Gradient Scaffold of Collagen–Hydroxyapatite for Osteochondral Regeneration. J. Tissue Eng..

[54] Terzi A., Storelli E., Bettini S., Sibillano T., Altamura D., Salvatore L., Madaghiele M., Romano A., Siliqi D., Ladisa M. (2018). Effects of Processing on Structural, Mechanical and Biological Properties of Collagen-Based Substrates for Regenerative Medicine. Sci. Rep..

[55] Gallo N., Natali M.L., Curci C., Picerno A., Gallone A., Vulpi M., Vitarelli A., Ditonno P., Cascione M., Sallustio F. (2021). Analysis of the Physico-Chemical, Mechanical and Biological Properties of Crosslinked Type-I Collagen from Horse Tendon: Towards the Development of Ideal Scaffolding Material for Urethral Regeneration. Materials.

[56] Yañez-Mó M., Barreiro O., Gonzalo P., Batista A., Megías D., Genís L., Sachs N., Sala-Valdés M., Alonso M.A., Montoya M.C. (2008). MT1-MMP Collagenolytic Activity Is Regulated through Association with Tetraspanin CD151 in Primary Endothelial Cells. Blood.

[57] Kwansa A.L., de Vita R., Freeman J.W. (2014). Mechanical Recruitment of N- and C-Crosslinks in Collagen Type I. Matrix Biol..

[58] Adhikari A.S., Chai J., Dunn A.R. (2011). Mechanical Load Induces a 100-Fold Increase in the Rate of Collagen Proteolysis by MMP-1. J. Am. Chem. Soc..

[59] Adhikari A.S., Glassey E., Dunn A.R. (2012). Conformational Dynamics Accompanying the Proteolytic Degradation of Trimeric Collagen I by Collagenases. J. Am. Chem. Soc..

[60] Maternini M., Guttadauro A., Mascagni D., Milito G., Stuto A., Renzi A., Ripamonti L., Bottini C., Nudo R., del Re L. (2019). Non Cross-Linked Equine Collagen (Salvecoll-E Gel) for Treatment of Complex Ano-Rectal Fistula. Asian J. Surg..

[61] Sandor M., Xu H., Connor J., Lombardi J., Harper J.R., Silverman R.P., McQuillan D.J. (2008). Host Response to Implanted Porcine-Derived Biologic Materials in a Primate Model of Abdominal Wall Repair. Tissue Eng. Part A.

[62] Bohn G., Liden B., Schultz G., Yang Q., Gibson D.J. (2016). Ovine-Based Collagen Matrix Dressing: Next-Generation Collagen Dressing for Wound Care. Adv. Wound Care.

[63] Neuber F. (1893). Fettransplantation bericht uber die verhandlungen der deutscht gesellsch chir. Zentralbl. Chir..

[64] Kontis T., Rivkin A. (2009). The History of Injectable Facial Fillers. Facial Plast. Surg..

[65] Cespedes R.D. (2000). Collagen Injection or Artificial Sphincter for Postprostatectomy Incontinence: Collagen. Urology.

[66] Cho K.-H., Uthaman S., Park I.-K., Cho C.-S. (2018). Injectable Biomaterials in Plastic and Reconstructive Surgery: A Review of the Current Status. Tissue Eng. Regen. Med..

[67] Inglefield C., Samuelson E.U., Landau M., DeVore D. (2018). Bio-Dermal Restoration With Rapidly Polymerizing Collagen: A Multicenter Clinical Study. Aesthetic Surg. J..

[68] Alnojeidi H., Kilani R.T., Ghahary A. (2022). Evaluating the Biocompatibility of an Injectable Wound Matrix in a Murine Model. Gels.

[69] Camilleri-Brennan J. (2014). Anal Injectables and Implantables for Faecal Incontinence. Fecal Incontinence—Causes, Management and Outcome.

[70] Thomas K., Engler A.J., Meyer G.A. (2015). Extracellular Matrix Regulation in the Muscle Satellite Cell Niche. Connect. Tissue Res..

[71] Lynn A.K., Yannas I.V., Bonfield W. (2004). Antigenicity and Immunogenicity of Collagen. J. Biomed. Mater. Res. B Appl. Biomater..

[72] Ellingsworth L.R., de Lustro F., Brennan J.E., Sawamura S., Mc Pherson J. (1986). The Human Immune Response to Reconstituted Bovine Collagen. J. Immunol..

[73] Charriere G., Bejot M., Schnitzler L., Ville G., Hartmann D.J. (1989). Reactions to a Bovine Collagen Implant: Clinical and Immunologic Study in 705 Patients. J. Am. Acad. Dermatol..

[74] Aamodt J.M., Grainger D.W. (2016). Extracellular Matrix-Based Biomaterial Scaffolds and the Host Response. Biomaterials.

[75] Lemperle G., Morhenn V., Charrier U. (2003). Human Histology and Persistence of Various Injectable Filler Substances for Soft Tissue Augmentation. Aesthetic Plast. Surg..

[76] Narins R.S., Brandt F., Leyden J., Lorenc Z.P., Rubin M., Smith S. (2003). A Randomized, Double-Blind, Multicenter Comparison of the Efficacy and Tolerability of Restylane versus Zyplast for the Correction of Nasolabial Folds. Dermatol. Surg..

[77] Solomon P., Sklar M., Zener R. (2012). Facial Soft Tissue Augmentation with Artecoll^®^: A Review of Eight Years of Clinical Experience in 153 Patients. Can. J. Plast. Surg..

[78] Lemperle G., Romani J.J., Busso M. (2003). Soft Tissue Augmentation With Artecoll: 10-Year History, Indications, Techniques, and Complications. Dermatol. Surg..

[79] Cohen S.R., Holmes R.E. (2004). Artecoll: A Long-Lasting Injectable Wrinkle Filler Material: Report of a Controlled, Randomized, Multicenter Clinical Trial of 251 Subjects. Plast. Reconstr. Surg..

[80] Haneke E. (2004). Polymethyl Methacrylate Microspheres in Collagen. Semin. Cutan. Med. Surg..

[81] Kim K.J., Lee H.W., Lee M.W., Choi J.H., Moon K.C., Koh J.K. (2004). Artecoll Granuloma: A Rare Adverse Reaction Induced by Microimplant in the Treatment of Neck Wrinkles. Dermatol. Surg..

[82] Rullan P.P. (2004). Soft Tissue Augmentation Using Artecoll: A Personal Experience. Facial Plast. Surg..

[83] Thaler M.P., Ubogy Z.I. (2005). Artecoll: The Arizona Experience and Lessons Learned. Dermatol. Surg..

[84] Solomon P., Ng C.L., Kerzner J., Rival R. (2021). Facial Soft Tissue Augmentation with Bellafill: A Review of 4 Years of Clinical Experience in 212 Patients. Plast. Surg..

[85] Cohen S.R., Berner C.F., Busso M., Gleason M.C., Hamilton D., Holmes R.E., Romano J.J., Rullan P.P., Thaler M.P., Ubogy Z. (2006). ArteFill: A Long-Lasting Injectable Wrinkle Filler Material—Summary of the U.S. Food and Drug Administration Trials and a Progress Report on 4- to 5-Year Outcomes. Plast. Reconstr. Surg..

[86] Moon S.H., Lee Y.J., Rhie J.W., Suh D.S., Oh D.Y., Lee J.H., Kim Y.J., Kim S.M., Jun Y.J. (2015). Comparative Study of the Effectiveness and Safety of Porcine and Bovine Atelocollagen in Asian Nasolabial Fold Correction. J. Plast. Surg. Hand Surg..

[87] Lee J.H., Choi Y.S., Kim S.M., Kim Y.J., Rhie J.W., Jun Y.J. (2014). Efficacy and Safety of Porcine Collagen Filler for Nasolabial Fold Correction in Asians: A Prospective Multicenter, 12 Months Follow-up Study. J. Korean Med. Sci..

[88] Hirche C., Senghaas A., Fischer S., Hollenbeck S.T., Kremer T., Kneser U. (2016). Novel Use of a Flowable Collagen-Glycosaminoglycan Matrix (Integra^TM^ Flowable Wound Matrix) Combined with Percutaneous Cannula Scar Tissue Release in Treatment of Post-Burn Malfunction of the Hand—A Preliminary 6 Month Follow-Up. Burns.

[89] Kligman A.M., Armstrong R.C. (1986). Histologic Respose to Intradermal Zyderm and Zyplast (Glutaraldehyde Cross-Linked) Collagen in Humans. J. Dermatol. Surg. Oncol..

[90] Stegman S.J., Chu S., Bensch K., Armstrong R. (1987). A Light and Electron Microscopic Evaluation of Zyderm Collagen and Zyplast Implants in Aging Human Facial Skin: A Pilot Study. Arch. Dermatol..

[91] Elson M.L. (1988). Clinical Assessment of Zyplast Implant: A Year of Experience for Soft Tissue Contour Correction. J. Am. Acad. Dermatol..

[92] Matti B.A., Nicolle F.v. (1990). Clinical Use of Zyplast in Correction of Age- and Disease-Related Contour Deficiencies of the Face. Aesthetic Plast. Surg..

[93] Cooperman L.S., Mackinnon V., Bechler G., Pharriss B.B. (1985). Injectable Collagen: A Six-Year Clinical Investigation. Aesthetic Plast. Surg..

[94] Castrow F.F., Krull E.A. (1983). Injectable Collagen Implant—Update. J. Am. Acad. Dermatol..

[95] Downie J., Mao Z., Rachel Lo T.W., Barry S., Bock M., Siebert J.P., Bowman A., Ayoub A. (2009). A Double-Blind, Clinical Evaluation of Facial Augmentation Treatments: A Comparison of PRI 1, PRI 2, Zyplast^®^ and Perlane^®^. J. Plast. Reconstr. Aesthetic Surg..

[96] Baumann L.S., Shamban A.T., Lupo M.P., Monheit G.D., Thomas J.A., Murphy D.K., Walker P.S. (2007). Comparison of Smooth-Gel Hyaluronic Acid Dermal Fillers with Cross-Linked Bovine Collagen: A Multicenter, Double-Masked, Randomized, within-Subject Study. Dermatol. Surg..

[97] Nevins M., Giannobile W.V., McGuire M.K., Kao R.T., Mellonig J.T., Hinrichs J.E., McAllister B.S., Murphy K.S., McClain P.K., Nevins M.L. (2005). Platelet-Derived Growth Factor Stimulates Bone Fill and Rate of Attachment Level Gain: Results of a Large Multicenter Randomized Controlled Trial. J. Periodontol..

[98] Nevins A., Crespi P. (1998). A Clinical Study Using the Collagen Gel Zyplast in Endodontic Treatment. J. Endod..

[99] Gadomski B.C., Labus K.M., Puttlitz C.M., McGilvray K.C., Regan D.P., Nelson B., Seim H.B., Easley J.T. (2021). Evaluation of Lumbar Spinal Fusion Utilizing Recombinant Human Platelet Derived Growth Factor-B Chain Homodimer (RhPDGF-BB) Combined with a Bovine Collagen/β-Tricalcium Phosphate (β-TCP) Matrix in an Ovine Model. JOR Spine.

[100] Scott R.T., McAlister J.E., Rigby R.B. (2018). Allograft Bone: What Is the Role of Platelet-Derived Growth Factor in Hindfoot and Ankle Fusions. Clin. Podiatr. Med. Surg..

[101] Daniels T.R., Anderson J., Swords M.P., Maislin G., Donahue R., Pinsker E., Quiton J.D. (2019). Recombinant Human Platelet–Derived Growth Factor BB in Combination with a Beta-Tricalcium Phosphate (RhPDGF-BB/β-TCP)-Collagen Matrix as an Alternative to Autograft. Foot Ankle Int..

[102] DiGiovanni C.W., Lin S.S., Baumhauer J.F., Daniels T., Younger A., Glazebrook M., Anderson J., Anderson R., Evangelista P., Lynch S.E. (2013). Recombinant Human Platelet-Derived Growth Factor-BB and Beta-Tricalcium Phosphate (RhPDGF-BB/β-TCP): An Alternative to Autogenous Bone Graft. J. Bone Jt. Surg..

[103] Daniels T., DiGiovanni C., Lau J.T.C., Wing K., Alastair Y. (2010). Prospective Clinical Pilot Trial in a Single Cohort Group of RhPDGF in Foot Arthrodeses. Foot Ankle Int..

[104] Abidi N.A., Younger A., Digiovanni C.W. (2012). Role of Platelet-Derived Growth Factor in Hindfoot Fusion. Tech. Foot Ankle Surg..

[105] Hollinger J.O., Hart C.E., Hirsch S.N., Lynch S., Friedlaender G.E. (2008). Recombinant Human Platelet-Derived Growth Factor: Biology and Clinical Applications. J. Bone Jt. Surg..

[106] DiGiovanni C.W., Petricek J.M. (2010). The Evolution of RhPDGF-BB in Musculoskeletal Repair and Its Role in Foot and Ankle Fusion Surgery. Foot Ankle Clin..

[107] Digiovanni C.W., Lin S., Pinzur M. (2012). Recombinant Human PDGF-BB in Foot and Ankle Fusion. Expert Rev. Med. Devices.

[108] DiGiovanni C.W., Baumhauer J., Lin S.S., Berberian W.S., Flemister A.S., Enna M.J., Evangelista P., Newman J. (2011). Prospective, Randomized, Multi-Center Feasibility Trial of RhPDGF-BB versus Autologous Bone Graft in a Foot and Ankle Fusion Model. Foot Ankle Int..

[109] DiGiovanni C.W., Lin S.S., Daniels T.R., Glazebrook M., Evangelista P., Donahue R., Beasley W., Baumhauer J.F. (2016). The Importance of Sufficient Graft Material in Achieving Foot or Ankle Fusion. J. Bone Jt. Surg. Am. Vol..

[110] Solchaga L.A., Daniels T., Roach S., Beasley W., Snel L.B. (2013). Effect of Implantation of Augment^®^ Bone Graft on Serum Concentrations of Platelet-Derived Growth Factors: A Pharmacokinetic Study. Clin. Drug Investig..

[111] Perrien D.S., Young C.S., Alvarez-Urena P.P., Dean D.D., Lynch S.E., Hollinger J.O. (2013). Percutaneous Injection of Augment Injectable Bone Graft (RhPDGF-BB and β-Tricalcium Phosphate [β-TCP]/Bovine Type i Collagen Matrix) Increases Vertebral Bone Mineral Density in Geriatric Female Baboons. Spine J..

[112] de Luca P., Colombini A., Carimati G., Beggio M., de Girolamo L., Volpi P. (2019). Intra-Articular Injection of Hydrolyzed Collagen to Treat Symptoms of Knee Osteoarthritis. A Functional in Vitro Investigation and a Pilot Retrospective Clinical Study. J. Clin. Med..

[113] Kimura M., Nito T., Imagawa H., Tayama N., Chan R.W. (2008). Collagen Injection as a Supplement to Arytenoid Adduction for Vocal Fold Paralysis. Ann. Otol. Rhinol. Laryngol..

[114] Kimura M., Nito T., Sakakibara K.I., Tayama N., Niimi S. (2008). Clinical Experience with Collagen Injection of the Vocal Fold: A Study of 155 Patients. Auris Nasus Larynx.

[115] Stojkovic S.G., Lim M., Burke D., Finan P.J., Sagar P.M. (2006). Intra-Anal Collagen Injection for the Treatment of Faecal Incontinence. Br. J. Surg..

[116] Jamal N., Mundi J., Chhetri D.K. (2014). Higher Risk of Superficial Injection during Injection Laryngoplasty in Women. Am. J. Otolaryngol..

[117] Ford C.N., Bless D.M., Loftus J.M. (1992). Role of Injectable Collagen in the Treatment of Glottic Insufficiency: A Study of 119 Patients. Ann. Otol. Rhinol. Laryngol..

[118] Hoffman H., McCabe D., McCulloch T., Jin S.M., Karnell M. (2002). Laryngeal Collagen Injection as an Adjunct to Medialization Laryngoplasty. Laryngoscope.

[119] Luu Q., Tsai V., Mangunta V., Berke G.S., Chhetri D.K. (2007). Safety of Percutaneous Injection of Bovine Dermal Crosslinked Collagen for Glottic Insufficiency. Otolaryngol. Head Neck Surg..

[120] Kamer F.M., Churukian M.M. (1984). Clinical Use of Injectable Collagen. Arch. Otolaryngol..

[121] Homma Y., Kawabe K., Kageyama S., Koiso K., Akaza H., Kakizoe T., Koshiba K., Yokoyama E., Aso Y. (1996). Injection of Glutaraldehyde Cross-Linked Collagen for Urinary Incontinence: Two-Year Efficacy by Self-Assessment. Int. J. Urol..

[122] Elsergany R., Elgamasy A.N., Ghoniem G.M. (1998). Transurethral Collagen Injection for Female Stress Incontinence. Int. Urogynecol. J..

[123] Cespedes R.D., Leng W.W., McGuire E.J. (1999). Collagen Injection Therapy for Postprostatectomy Incontinence. Urology.

[124] Corcos J., Fournier C. (1999). Periurethral Collagen Injection for the Treatment of Female Stress Urinary Incontinence: 4-Year Follow-up Results. Urology.

[125] Gorton E., Stanton S., Monga A., Wiskind A.K., Lentz G.M., Bland D.R. (1999). Periurethral Collagen Injection: A Long-Term Follow-up Study. BJU Int..

[126] Vandenbulcke L., Lapage K.G., Vanderstraeten K.V., de Somer F.M., de Hert S.G., Moerman A.T. (2017). Microvascular Reactivity Monitored with Near-Infrared Spectroscopy Is Impaired after Induction of Anaesthesia in Cardiac Surgery Patients. Eur. J. Anaesthesiol..

[127] Marx G., Zacharowski K., Ichai C., Asehnoune K., Černý V., Dembinski R., Ferrer Roca R., Fries D., Molnar Z., Rosenberger P. (2021). Efficacy and Safety of Early Target-Controlled Plasma Volume Replacement with a Balanced Gelatine Solution versus a Balanced Electrolyte Solution in Patients with Severe Sepsis/Septic Shock: Study Protocol, Design, and Rationale of a Prospective, Randomized, Controlled, Double-Blind, Multicentric, International Clinical Trial: GENIUS—Gelatine Use in ICU and Sepsis. Trials.

[128] Hamraoui K., Ernst S.M.P.G., van Dessel P.F.H.M., Kelder J.C., ten Berg J.M., Suttorp J., Jaarsma W., Plokker H.W. (2002). Efficacy and Safety of Percutaneous Treatment of Iatrogenic Femoral Artery Pseudoaneurysm by Biodegradable Collagen Injection. J. Am. Coll. Cardiol..

[129] Majdalany B.S., Willatt J., Beecham Chick J.F., Srinivasa R.N., Saad W.A. (2017). Fibrillar Collagen Injection for Organ Protection during Thermal Ablation of Hepatic Malignancies. Diagn. Interv. Radiol..

[130] Nitecka-Buchta A., Walczynska-Dragon K., Batko-Kapustecka J., Wieckiewicz M. (2018). Comparison between Collagen and Lidocaine Intramuscular Injections in Terms of Their Efficiency in Decreasing Myofascial Pain within Masseter Muscles: A Randomized, Single-Blind Controlled Trial. Pain Res. Manag..

[131] Giovannangeli F., Bizzi E., Massafra U., Vacca F., Tormenta S., Migliore A. (2016). Intra-Articular Administration of MD-HIP in 24 Patients Affected by Symptomatic Hip Osteoarthritis—A 24-Month Cohort Study. Physiol. Regul. Med..

[132] Guitart Vela J., Folch Ibanez J. (2016). Collagen MDs for Chronic Pain. Efficacy and Tolerability in Chronic Treatment in 124 Patients. Physiol. Regul. Med..

[133] Reshkova V., Rashkov R., Nestorova R. (2016). Efficacy and Safety Evaluation of GUNA Collagen MDs Injections in Knee Osteoarthritis—A Case Series of 30 Patients. Physiol. Regul. Med..

[134] Pavelka K., Jarosova H., Milani L., Prochazka Z., Kostiuk P., Kotlarova L., Meroni A.M., Slíva J. (2019). Efficacy and Tolerability of Injectable Collagen-Containing Products in Comparison to Trimecaine in Patients with Acute Lumbar Spine Pain (Study FUTURE-MD-Back Pain). Physiol. Res..

[135] Massulo C. (2016). Injectable GUNA Collagen Medical Devices in Functional Recovery from Sport. Physiol. Regul. Med..

[136] Staňa J. (2016). 3 Years in Luhačovice Spa with Collagen Medical Devices Injections in the Treatment of Piriformis Syndrome. Physiol. Regul. Med..

[137] Alfieri N. (2016). MD-Muscle in the Management of Myofascial Pain Syndrome. Physiol. Regul. Med..

[138] Heng C.H.Y., Snow M., Dave L.Y.H. (2021). Single-Stage Arthroscopic Cartilage Repair With Injectable Scaffold and BMAC. Arthrosc. Tech..

[139] Kim M.S., Chun C.H., Wang J.H., Kim J.G., Kang S.B., Yoo J.D., Chon J.G., Kim M.K., Moon C.W., Chang C.B. (2020). Microfractures Versus a Porcine-Derived Collagen-Augmented Chondrogenesis Technique for Treating Knee Cartilage Defects: A Multicenter Randomized Controlled Trial. Arthrosc. J. Arthrosc. Relat. Surg..

[140] Lee H.S., Oh K.J., Moon Y.W., In Y., Lee H.J., Kwon S.Y. (2021). Intra-Articular Injection of Type I Atelocollagen to Alleviate Knee Pain: A Double-Blind, Randomized Controlled Trial. Cartilage.

[141] Kim J.H., Kim D.J., Lee H.J., Kim B.K., Kim Y.S. (2020). Atelocollagen Injection Improves Tendon Integrity in Partial-Thickness Rotator Cuff Tears: A Prospective Comparative Study. Orthop. J. Sports Med..

[142] Volpi P., Zini R., Erschbaumer F., Beggio M., Busilacchi A., Carimati G. (2021). Effectiveness of a Novel Hydrolyzed Collagen Formulation in Treating Patients with Symptomatic Knee Osteoarthritis: A Multicentric Retrospective Clinical Study. Int. Orthop..

[143] Mariconti P. (2016). Usefulness of GUNA Collagen Medical Devices in the Treatment of Knee Pain. Physiol. Regul. Med..

[144] Martin Martin L.S., Massafra U., Bizzi E., Migliore A. (2016). A Double Blind Randomized Active-Controlled Clinical Trial on the Intra-Articular Use of Md-Knee versus Sodium Hyaluronate in Patients with Knee Osteoarthritis (“Joint”). BMC Musculoskelet. Disord..

[145] Uroz N.Z. (2016). Collagen Medical Device Infiltrations in Shoulder Pathologies. Calcific Supraspinatus Tendinitis. Physiol. Regul. Med..

[146] Nestorova R., Rashkov R., Petranova T. (2016). Clinical and Sonographic Assessment of the Effectiveness of GUNA Collagen MDs Injections in Patients with Partial Thickness Tear of the Rotator Cuff. Physiol. Regul. Med..

[147] Wolkow N., Jakobiec F.A., Dryja T.P., Lefebvre D.R. (2018). Mild Complications or Unusual Persistence of Porcine Collagen and Hyaluronic Acid Gel Following Periocular Filler Injections. Ophthalmic Plast. Reconstr. Surg..

[148] Braun M., Braun S. (2008). Nodule Formation Following Lip Augmentation Using Porcine Collagen-Derived Filler. J. Drugs Dermatol..

[149] Narins R.S., Brandt F.S., Lorenc Z.P., Maas C.S., Monheit G.D., Smith S.R. (2008). Twelve-Month Persistency of a Novel Ribose-Cross-Linked Collagen Dermal Filler. Dermatol. Surg..

[150] Pollack S.v. (1990). Silicone, Fibrel, and Collagen Implantation for Facial Lines and Wrinkles. J. Dermatol. Surg. Oncol..

[151] Mittelman H. (1988). Fibrel: A Dermal Implant Comparison With Collagen Implants. Arch. Otolaryngol. Head Neck Surg..

[152] Denton A.B., Shoman N. (2010). Porcine Collagen: Evolence. Office-Based Cosmetic Procedures and Techniques.

[153] Samalavicius N.E., Kavaliauskas P., Dulskas A. (2020). Permacol^TM^ Collagen Paste Injection for the Treatment of Complex Anal Fistula—A Video Vignette. Color. Dis..

[154] Pescatori M. (2017). Permacol^TM^ Collagen Paste for Treating a Rectovaginal Fistula Following Anterior Rectal Prolapsectomy. Tech. Coloproctol..

[155] Harran N., Herold J., Bentley A., Bebington B.D. (2017). Efficacy of Porcine Dermal Collagen (PermacolTM) Injection for Passive Faecal Incontinence in a Dedicated Colorectal Unit at the Wits Donald Gordon Medical Centre. S. Afr. J. Surg..

[156] Giordano P., Sileri P., Buntzen S., Stuto A., Nunoo-Mensah J., Lenisa L., Singh B., Thorlacius-Ussing O., Griffiths B., Ziyaie D. (2016). A Prospective Multicentre Observational Study of Permacol^TM^ Collagen Paste for Anorectal Fistula: Preliminary Results. Color. Dis..

[157] Sileri P., Franceschilli L., del Vecchio Blanco G., Stolfi V.M., Angelucci G.P., Gaspari A.L. (2011). Porcine Dermal Collagen Matrix Injection May Enhance Flap Repair Surgery for Complex Anal Fistula. Int. J. Color. Dis..

[158] Milito G., Cadeddu F. (2009). Conservative Treatment for Anal Fistula: Collagen Matrix Injection. J. Am. Coll. Surg..

[159] Harper C. (2001). Permacol: Clinical Experience with a New Biomaterial. Hosp. Med..

[160] Micarelli A., Viziano A., Granito I., Antonuccio G., Felicioni A., Loberti M., Carlino P., Micarelli R.X., Alessandrini M. (2021). Combination of In-Situ Collagen Injection and Rehabilitative Treatment in Long-Lasting Facial Nerve Palsy: A Pilot Randomized Controlled Trial. Eur. J. Phys. Rehabil. Med..

[161] del Carpio-Orantes L., García-Méndez S., Sánchez-Díaz J.S., Aguilar-Silva A., Contreras-Sánchez E.R., Hernández S.N.H. (2021). Use of Fibroquel^®^ (Polymerized Type I Collagen) in Patients with Hypoxemic Inflammatory Pneumonia Secondary to COVID-19 in Veracruz, Mexico. J. Anesth. Crit. Care.

[162] Méndez-Flores S., Priego-Ranero Á., Azamar-Llamas D., Olvera-Prado H., Rivas-Redonda K.I., Ochoa-Hein E., Perez-Ortiz A., Rendón-Macías M.E., Rojas-Castañeda E., Urbina-Terán S. (2022). Effect of Polymerised Type I Collagen on Hyperinflammation of Adult Outpatients with Symptomatic COVID-19. Clin. Transl. Med..

[163] Sparavigna A., Tateo A., Inselvini E., Tocchio M., Orlandini M.C., Botali G. (2017). Anti-Age Activity and Tolerance Evaluation of Collagen Micro-Injection Treatment Associated to Topical Application of a Cosmetic Formulation (Investigator-Initiated Multicentre Trial). J. Clin. Exp. Dermatol. Res..

[164] Gkouvi A., Nicolaidou E., Corbo A., Selvaggi G., Tsimpidakis A., Mastraftsi S., Gregoriou S. (2021). Heterologous Type i Collagen as an Add-on Therapy to Narrowband Ultraviolet b for the Treatment of Vitiligo: A Pilot Study. J. Clin. Aesthetic Dermatol..

[165] Gkouvi A., Corbo A., Gregoriou S. (2020). Treatment of Male Genital Lichen Sclerosus with Heterologous Type I Collagen. Clin. Exp. Dermatol..

[166] Klewin-Steinböck S., Nowak-Terpi3owska A., Adamski Z., Grocholewicz K., Wyganowska-Swiatkowska M. (2021). Effect of Injectable Equine Collagen Type I on Metabolic Activity and Apoptosis of Gingival Fibroblasts. Adv. Dermatol. Allergol..

[167] Wyganowska-Swiatkowska M., Duda-Sobczak A., Corbo A., Matthews-Brzozowska T. (2020). Atelocollagen Application in Human Periodontal Tissue Treatment—A Pilot Study. Life.

[168] Burres S. (2001). Soft-Tissue Augmentation with Fascian. Clin. Plast. Surg..

[169] Bauman L. (2004). CosmoDerm/CosmoPlast (Human Bioengineered Collagen) for the Aging Face. Facial Plast. Surg..

[170] Fagien S., Elson M.L. (2001). Facial Soft-Tissue Augmentation with Allogeneic Human Tissue Collagen Matrix (Dermalogen and Dermaplant). Clin. Plast. Surg..

[171] Burres S. (2004). Fascian. Facial Plast. Surg..

[172] Maloney B.P., Murphy B.A., Cole H.P. (2004). Cymetra. Facial Plast. Surg..

[173] Anderson T.D., Sataloff R.T. (2004). Complications of Collagen Injection of the Vocal Fold: Report of Several Unusual Cases and Review of the Literature. J. Voice.

[174] Bock J.M., Lee J.H., Robinson R.A., Hoffman H.T. (2007). Migration of Cymetra After Vocal Fold Injection for Laryngeal Paralysis. Laryngoscope.

[175] Karpenko A.N., Meleca R.J., Dworkin J.P., Stachler R.J. (2003). Cymetra Injection for Unilateral Vocal Fold Paralysis. Ann. Otol. Rhinol. Laryngol..

[176] Douglas R.S., Donsoff I., Cook T., Shorr N. (2004). Collagen Fillers in Facial Aesthetic Surgery. Facial Plast. Surg..

[177] Homicz M.R., Watson D. (2004). Review of Injectable Materials for Soft Tissue Augmentation. Facial Plast. Surg..

[178] Rinaldi F., Pinto D., Trink A., Giuliani G., Sparavigna A. (2021). In Vitro and in Vivo Evaluation on the Safety and Efficacy of a Brand-New Intracutaneous Filler with A1-R-Collagen. Clin. Cosmet. Investig. Dermatol..

[179] Lombardi F., Palumbo P., Augello F.R., Giusti I., Dolo V., Guerrini L., Cifone M.G., Giuliani M., Cinque B. (2020). Type I Collagen Suspension Induces Neocollagenesis and Myodifferentiation in Fibroblasts in Vitro. Biomed. Res. Int..

[180] Keefe J., Wauk L., Chu S., DeLustro F. (1992). Clinical Use of Injectable Bovine Collan: A Decade of OExperience. Clin. Mater..

[181] Randelli F., Menon A., Via A.G., Mazzoleni M., Sciancalepore F., Brioschi M., Gagliano N. (2018). Effect of a Collagen-Based Compound on Morpho-Functional Properties of Cultured Human Tenocytes. Cells.

[182] Furuzawa-Carballeda J., Lima G., Llorente L., Nuñez-Álvarez C., Ruiz-Ordaz B.H., Echevarría-Zuno S., Hernández-Cuevas V. (2012). Polymerized-Type I Collagen Downregulates Inflammation and Improves Clinical Outcomes in Patients with Symptomatic Knee Osteoarthritis Following Arthroscopic Lavage: A Randomized, Double-Blind, and Placebo-Controlled Clinical Trial. Sci. World J..

[183] Corrado B., Bonini I., Alessio Chirico V., Rosano N., Gisonni P. (2020). Use of Injectable Collagen in Partial-Thickness Tears of the Supraspinatus Tendon: A Case Report. Oxf. Med. Case Rep..

[184] Kim M., Choi Y.S., You M.-W., Kim J.S., Young K.W. (2016). Sonoelastography in the Evaluation of Plantar Fasciitis Treatment 3-Month Follow-Up After Collagen Injection. Ultrasound Q..

[185] Martins S.B., Oliveira E., Castro R.A., Sartori M.G., Baracat E.C., Lima G.R., Girao M.J. (2007). Clinical and Urodynamic Evaluation in Women with Stress Urinary Incontinence Treated by Periurethral Collagen Injection. Int. Braz. J. Urol..

[186] Oremus M., Tarride J.-E. (2010). An Economic Evaluation of Surgery versus Collagen Injection for the Treatment of Female Stress Urinary Incontinence. Can. J. Urol..

[187] Winters J.C., Chiverton A., Scarpero H.M., Prats L.J. (2000). Collagen injection therapy in elderly women: Long-term results and patient satisfaction. Urology.

[188] Groutz A., Blaivas J.G., Kesler S.S., Weiss J.P., Chaikin D.C. (2000). Female urology outcome results of transurethral collagen injection for female stress incontinence: Assessment by urinary incontinence score. J. Urol..

[189] Bomalaski M.D., Bloom D.A., McGuire E.J., Panzl A. (1996). Glutaraldehyde cross-linked collagen in the treatment of urinary incontinence in children. J. Urol..

[190] Kassouf W., Capolicchio G., Berardinucci G., Corcos J. (2001). Collagen injection for treatment of urinary incontinence in children. J. Urol..

[191] Faerber G.J., Richardson T.D. (1997). Long-Term Results of Transurethral Collagen Injection in Men with Intrinsic Sphincter Deficiency.

[192] Faerber G.J., Belville W.D., Ohl D.A., Plata A. (1998). Comparison of Transurethral versus Periurethral Collagen Injection in Women with Intrinsic Sphincter Deficiency. Tech. Urol..

[193] Richardson T.D., Kennelly M.J., Faerber G.J. (1995). Endoscopic injection of clutaraldehyde cross-linked collagen for the treatment of intrinsic sphincter deficiency in women. Urology.

[194] Smith D.N., Appell R.A., Rackley R.R., Winters J.C. (1998). Collagen Injection Therapy for Post-Prostatectomy Incontinence. J. Urol..

[195] Klutke C.G., Tiemann D.D., Nadler R.B., Andriole G.L. (1996). Antegrade Collagen Injection for Stress Incontinence after Radical Prostatectomy: Technique and Early Results.

[196] Appell R.A. (1994). Collagen Injection Therapy for Urinary Incontinence. Urol. Clin. N. Am..

[197] Appell R.A., Vasavada S.P., Rackley R.R., Winters J.C. (1996). Percutaneous antegrade collagen injection therapy for urinary incontinence following radical prostatectomy. Urology.

[198] Nagai A., Nasu Y., Watanabe M., Tsugawa M., Iguchi H., Kumon H. (2004). Analysis of Retrograde Ejaculation Using Color Doppler Ultrasonography before and after Transurethral Collagen Injection. Int. J. Impot. Res..

[199] Spiegel J.R., Sataloff R.T., Gould W.J. (1987). The Treatment of Vocal Fold Paralysis with Injectable Collagen: Clinical Concerns. J. Voice.

[200] Remacle M., Lawson G. (2007). Results with Collagen Injection into the Vocal Folds for Medialization. Curr. Opin. Otolaryngol. Head Neck Surg.

[201] Remacle M., Hamoir M., Marbaix E. (1990). Gax-Collagen Injection to Correct Aspiration Problems after Subtotal Laryngectomy. Laryngoscope.

[202] Ford C.N., Bless D.M. (1993). Selected Problems Treated by Vocal Fold Injection of Collagen. Am. J. Otolaryngol..

[203] Lundy D.S., Casiano R.R., McClinton M.E., Xue J.W. (2003). Early Results of Transcutaneous Injection Laryngoplasty with Micronized Acellular Dermis Versus Type-I Thyroplasty for Glottic Incompetence Dysphonia Due to Unilateral Vocal Fold Paralysis. J. Voice.

[204] Rydningen M., Dehli T., Wilsgaard T., Rydning A., Kumle M., Lindsetmo R.O., Norderval S. (2017). Sacral Neuromodulation Compared with Injection of Bulking Agents for Faecal Incontinence Following Obstetric Anal Sphincter Injury—A Randomized Controlled Trial. Color. Dis..

[205] Brown S.A., Rohrich R.J., Baumann L., Brandt F.S., Fagien S., Glazer S., Kenkel J.M., Lowe N.J., Monheit G.D., Narins R.S. (2011). Subject Global Evaluation and Subject Satisfaction Using Injectable Poly-l-Lactic Acid versus Human Collagen for the Correction of Nasolabial Fold Wrinkles. Plast. Reconstr. Surg..

[206] Narins R.S., Baumann L., Brandt F.S., Fagien S., Glazer S., Lowe N.J., Monheit G.D., Rendon M.I., Rohrich R.J., Werschler W.P. (2010). A Randomized Study of the Efficacy and Safety of Injectable Poly-L-Lactic Acid versus Human-Based Collagen Implant in the Treatment of Nasolabial Fold Wrinkles. J. Am. Acad. Dermatol..

[207] Draelos Z. (2010). Case Study of Dermicol-P35 Used in Patient with Past Hypersensitivity to Crosslinked Bovine Collagen Dermal Filler. Dermatol. Surg..

[208] Narins R.S., Brandt F.S., Lorenc Z.P., Maas C.S., Monheit G.D., Smith S.R., Mcintyre S. (2007). A Randomized, Multicenter Study of the Safety and Efficacy of Dermicol-P35 and Non-Animal-Stabilized Hyaluronic Acid Gel for the Correction of Nasolabial Folds. Dermatol. Surg..

[209] Cassuto D. (2009). The Use of Dermicol-P35 Dermal Filler for Nonsurgical Rhinoplasty. Aesthetic Surg. J..

[210] Smith K.C. (2009). Repair of Acne Scars With Dermicol-P35. Aesthetic Surg. J..

[211] Horvath K. The Effect of GUNA-MDs in the Therapy Resistant Facial Paresis. Proceedings of the International Congress of PRM, Low doses therapies.

[212] Ding L., Yan G., Wang B., Xu L., Gu Y., Ru T., Cui X., Lei L., Liu J., Sheng X. (2018). Transplantation of UC-MSCs on Collagen Scaffold Activates Follicles in Dormant Ovaries of POF Patients with Long History of Infertility. Sci. China Life Sci..

[213] Bechara C.F., Annambhotla S., Lin P.H. (2010). Access Site Management with Vascular Closure Devices for Percutaneous Transarterial Procedures. J. Vasc. Surg..

[214] Awad S., Dharmavaram S., Wearn C.S., Dube M.G., Lobo D.N. (2012). Effects of an Intraoperative Infusion of 4 Succinylated Gelatine (Gelofusine^®^) and 6 Hydroxyethyl Starch (Voluven^®^) on Blood Volume. Br. J. Anaesth..

[215] Fisher G.J., Varani J., Voorhees J.J. (2008). Looking Older Fibroblast Collapse and Therapeutic Implications. Arch. Dermatol..

[216] Watson W., Kaye R.L., Klein A., Stegman S. (1983). Injectable Collagen: A Clinical Overview. Cutis.

[217] Nijhawan R.I., Cohen J.I., Kaufman J. (2008). Persistent Erythema After Human Collagen Filler Injections. Cosmet. Dermatol..

[218] Pereira D., Peleteiro B., Araújo J., Branco J., Santos R.A., Ramos E. (2011). The Effect of Osteoarthritis Definition on Prevalence and Incidence Estimates: A Systematic Review. Osteoarthr. Cartil..

[219] Kapoor M., Martel-Pelletier J., Lajeunesse D., Pelletier J.-P., Fahmi H. (2011). Role of Proinflammatory Cytokines in the Pathophysiology of Osteoarthritis. Nat. Rev. Rheumatol..

[220] Cui A., Li H., Wang D., Zhong J., Chen Y., Lu H. (2020). Global, Regional Prevalence, Incidence and Risk Factors of Knee Osteoarthritis in Population-Based Studies. EClinicalMedicine.

[221] Meimandi-Parizi A., Oryan A., Moshiri A. (2013). Role of Tissue Engineered Collagen Based Tridimensional Implant on the Healing Response of the Experimentally Induced Large Achilles Tendon Defect Model in Rabbits: A Long Term Study with High Clinical Relevance. J. Biomed. Sci..

[222] Suh D.-S., Lee J.-K., Yoo J.-C., Woo S.-H., Kim G.-R., Kim J.-W., Choi N.-Y., Kim Y., Song H.-S. (2017). Atelocollagen Enhances the Healing of Rotator Cuff Tendon in Rabbit Model. Am. J. Sport. Med..

[223] Kirlum H.J., Stehr M., Dietz H.G. (2006). Late Obstruction after Subureteral Collagen Injection. Eur. J. Pediatr. Surg..

[224] Hartl D.M., Riquet M., Hans S., Laccourreye O., Vaissière J., Brasnu D.F. (2001). Objective Voice Analysis after Autologous Fat Injection for Unilateral Vocal Fold Paralysis. Ann. Otol. Rhinol. Laryngol..

[225] Hirano M., Mori K., Tanaka S., Fujita M. (1995). Vocal Function in Patients with Unilateral Vocal Fold Paralysis Before and After Silicone Injection. Acta. Otolaryngol..

[226] He X., Wang Q., Zhao Y., Zhang H., Wang B., Pan J., Li J., Yu H., Wang L., Dai J. (2020). Effect of Intramyocardial Grafting Collagen Scaffold With Mesenchymal Stromal Cells in Patients With Chronic Ischemic Heart Disease. JAMA Netw. Open.

[227] Swanson N.A., Stoner J.G., Siegle R.J., Solomon A.R. (1983). Treatment Site Reactions to Zyderm Collagen Implantation. J. Dermatol. Surg. Oncol..

[228] Elson M.L. (1989). The Role of Skin Testing in the Use of Collagen Injectable Materials. J. Dermatol. Surg. Oncol..

[229] Aragona F., D’Urso L., Marcolongo R. (1998). Immunologic Aspects of Bovine Injectable Collagen in Humans. Eur. Urol..

[230] Zhou Z., Chen X., Zhou X., Yang X., Lu D., Kang W., Feng X. (2019). Effects of Intraoperative Gelatin on Blood Viscosity and Oxygenation Balance. J. PeriAnesthesia Nurs..

[231] Lucey P., Goldberg D. (2014). Complications of Collagen Fillers. Facial Plast. Surg..

[232] Cooperman L., Michaeli D., Alto P., Francisco S. (1984). The Immunogenicity of Injectable Collagen. II. A Retrospective Review of Seventy-Two Tested and Treated Patients. J. Am. Acad. Dermatol..

[233] Lemperle G., Rullan P.P., Gauthier-Hazan N. (2006). Avoiding and Treating Dermal Filler Complications. Plast. Reconstr. Surg..

[234] Vanderveen E.E., McCoy J.P., Schade W., Kapur J.J., Hamilton T., Ragsdale C., Grekin R.C., Swanson N.A. (1986). The Association of HLA and Immune Responses to Bovine Collagen Implants. Arch. Dermatol..

[235] Klein A.W. (1989). In Favor of Double Testing. Dermatol. Surg..

[236] Morgan K. (1990). What Do Anti-Collagen Antibodies Mean?. Ann. Rheum. Dis..

[237] Holmes R., Kirk S., Tronci G., Yang X., Wood D. (2017). Influence of Telopeptides on the Structural and Physical Properties of Polymeric and Monomeric Acid-Soluble Type I Collagen. Mater. Sci. Eng. C.

[238] Steffen C., Timpl R., Wolff I. (1968). Immunogenicity and Specificity of Collagen. V. Demonstration of Three Different Antigenic Determinants on Calf Collagen. Immunology.

[239] Leonard M.P., Decter A., Mix L.W., Johnson H.W., Coleman G.U. (1996). Endoscopic Treatment of Vesicoureteral Reflux with Collagen: Preliminary Report and Cost Analysis. J. Urol..

[240] Brown J.A., Elliott D.S., Barrett D.M. (1998). Postprostatectomy Urinary Incontinence: A Comparison of the Cost of Conservative Versus Surgical Management. Urology.

